# Proteomic Analysis of Generative and Vegetative Nuclei Reveals Molecular Characteristics of Pollen Cell Differentiation in *Lily*

**DOI:** 10.3389/fpls.2021.641517

**Published:** 2021-06-07

**Authors:** Chen You, YuPing Zhang, ShaoYu Yang, Xu Wang, Wen Yao, WeiHuan Jin, Wei Wang, XiuLi Hu, Hao Yang

**Affiliations:** ^1^State Key Laboratory of Wheat and Maize Crop Science, College of Life Sciences, Henan Agricultural University, Zhengzhou, China; ^2^College of Life Science, Henan Normal University, Xinxiang, China; ^3^Key Laboratory of Plant Molecular Physiology, Institute of Botany, Chinese Academy of Sciences, Beijing, China

**Keywords:** cell differentiation, pollen, vegetative cell, generative cell, nuclear proteins, lily, proteomic

## Abstract

In plants, the cell fates of a vegetative cell (VC) and generative cell (GC) are determined after the asymmetric division of the haploid microspore. The VC exits the cell cycle and grows a pollen tube, while the GC undergoes further mitosis to produce two sperm cells for double fertilization. However, our understanding of the mechanisms underlying their fate differentiation remains limited. One major advantage of the nuclear proteome analysis is that it is the only method currently able to uncover the systemic differences between VC and GC due to GC being engulfed within the cytoplasm of VC, limiting the use of transcriptome. Here, we obtained pure preparations of the vegetative cell nuclei (VNs) and generative cell nuclei (GNs) from germinating lily pollens. Utilizing these high-purity VNs and GNs, we compared the differential nucleoproteins between them using state-of-the-art quantitative proteomic techniques. We identified 720 different amount proteins (DAPs) and grouped the results in 11 fate differentiation categories. Among them, we identified 29 transcription factors (TFs) and 10 cell fate determinants. Significant differences were found in the molecular activities of vegetative and reproductive nuclei. The TFs in VN mainly participate in pollen tube development. In comparison, the TFs in GN are mainly involved in cell differentiation and male gametogenesis. The identified novel TFs may play an important role in cell fate differentiation. Our data also indicate differences in nuclear pore complexes and epigenetic modifications: more nucleoporins synthesized in VN; more histone variants and chaperones; and structural maintenance of chromosome (SMC) proteins, chromatin remodelers, and DNA methylation-related proteins expressed in GN. The VC has active macromolecular metabolism and mRNA processing, while GC has active nucleic acid metabolism and translation. Moreover, the members of unfolded protein response (UPR) and programmed cell death accumulate in VN, and DNA damage repair is active in GN. Differences in the stress response of DAPs in VN vs. GN were also found. This study provides a further understanding of pollen cell differentiation mechanisms and also a sound basis for future studies of the molecular mechanisms behind cell fate differentiation.

## Introduction

The plant male gametophyte (pollen) development begins with a diploid microspore mother cell differentiation, which produces haploid gametes through meiosis and mitosis. This process involves a series of cell fate determination and differentiation (Borg et al., 2009). A key event in pollen development is the asymmetric division of the post-meiotic haploid microspore resulting in two distinct fate daughter cells, vegetative cell (VC) and generative cell (GC) (Rutley and Twell, 2015). The larger VC would exit the cell cycle, nourish the GC, and control the pollen tube’s development. The diminutive GC, engulfed in VC’s cytoplasm, would undergo further mitosis to give rise to two sperm cells (SCs). Once the mature pollens land on the stigma, the germinated pollen tube would deliver the two SCs to the ovule for double fertilization (Berger and Twell, 2011; Yang et al., 2016).

The cellular differentiation in pollen is accompanied by nuclear differentiation (She and Baroux, 2015). The vegetative cell nucleus (VN) disperses chromatin devoid of heterochromatin domains and lowers CG DNA methylation levels and histone modifications such as H3K9me2, H3K4me2, and H3K9ac. In contrast, the generative cell nucleus (GN) has highly condensed chromatin, higher CG methylation, and aforementioned histone modifications (Tanaka et al., 1998; Schoft et al., 2009; Houben et al., 2011). Interestingly, transposable element (TE)-derived small interfering RNAs could be activated and expressed in VN and then transferred to sperm nuclei (SN) to reinforce the genome stability of SN through silencing of complementary TEs in SN (Slotkin et al., 2009; Calarco et al., 2012). Remarkably, a growing body of research proved that histone programs are essential for cell identity establishment and differentiation by affecting gene expression (Henikoff and Smith, 2015). In lily, some histone variants are exclusively expressed in GN, such as gH2A, gH2B, gcH3, gH3, leH3, soH3-1, and soH3-2 (Ueda and Tanaka, 1994; Ueda et al., 2000; Sano and Tanaka, 2005; Okada et al., 2006b). Our previous research revealed that a total of nine histone variants, including five H1, two H2B, one H3, and one H4 variant, are specifically accumulated in GN of lily pollen (Yang et al., 2016). In Arabidopsis, different H3 variants have also been observed between VCs and sperms (Ingouff et al., 2007; Ingouff et al., 2010).

Transcriptomics analyses and genetic studies in Arabidopsis have identified a body of genes involved in pollen cell fate differentiation. Some genes are firstly expressed in the microspore and have roles in microtubule-dependent polarity establishment and cytokinesis, such as *GEM1* (*GEMINI POLLEN1*), *TUBG1/2* (*Gamma-tubulin 1/2*), *TIO* (*TWO-IN-ONE*), and *KINESIN-12A/B* (Park et al., 1998; Whittington et al., 2001; Twell et al., 2002; Pastuglia et al., 2006; Lee et al., 2007; Oh et al., 2010; Twell, 2011; Oh et al., 2012). Other genes are activated after the first microspore mitosis. The transcripts of these genes are differentially accumulated between VC and SC, mainly including transcription factors and some cell cycle-related genes. *F-box-Like 17* (*FBL17*) is transiently and exclusively expressed in the male germ cell. It could form a SCF^FBL17^ E3 ubiquitin ligase complex, which degrades specifically the cyclin-dependent kinase inhibitors KRP6 and KRP7 to ensure the production of two SCs. Conversely, KRP6/7 is always present in the VC and prevents VC cycle progression by inhibiting CDKA activity (Berger and Twell, 2011). Transcription factors DUO POLLEN1/3 (DUO1/3) and E2Fs are exclusively expressed in the male germ cell and involved in the regulation of male germline development through regulating some target genes, such as *CYCLINB1;1* (*CYCB1;1*), *Gamete Expressed 2* (*GEX2*), and *GCS1* (*Generative Cell Specific 1*) (Rotman et al., 2005; Lee et al., 2007; Brownfield et al., 2009; Borg et al., 2011; Zheng et al., 2011; Peters et al., 2017; Yao et al., 2018). Meanwhile, some specific genes in VC have also been identified, characterized by specific *cis*-regulatory elements (Rogers et al., 2001; Verelst et al., 2006). Although these essential genes have been identified, the mechanisms underlying pollen cell fate differentiation are still mostly unknown. Comprehensive protein and or gene landscape comparison between VC and GC can reveal the whole-cell internal molecular activity, give more clues, and shed light on the pollen cell fate differentiation mechanism. We have addressed to some extent this topic in the discussion section of this paper. However, the available data are quite limited. The nuclear proteomic analysis is currently the only method to uncover the systemic differences between VC and GC due to GC being engulfed within the cytoplasm of VC, limiting the use of transcriptome. Moreover, the major regulators of cell fate are in the nucleus, such as TFs, cell fate determinants, histone variants, and cell cycle regulatory proteins. A fundamental prerequisite for nuclear proteome analysis is the isolation of VN and GN with high purity. It is challenging to isolate VN and GN from tricellular pollen such as Arabidopsis pollen because GC’s mitosis occurs within the anther. In two-celled pollen such as that of lily, the mitosis of GC occurs during pollen-tube growth, making it relatively easy to isolate VN and GN.

In this study, we have successfully isolated a large amount of VNs and GNs with high purity from lily pollens by our previously established procedure (Yang et al., 2016). Using quantitative-based proteomic approaches, such as isobaric tags for relative and absolute quantification (iTRAQ), we identified 720 DAPs when searching against a custom-made lily protein database. We used phenotypic characterization, Gene Ontology (GO) functional, and Kyoto Encyclopedia of Genes and Genomes (KEGG) pathway enrichment analyses of DAPs and protein–protein interaction (PPI) network to evaluate the data. The results suggest significant differences in the molecular activities of vegetative and reproductive nuclei. Our study reveals the VC and GC’s nucleoprotein landscape for the first time, which is valuable for exploring the mechanisms underlying the pollen cell fate differentiation.

## Materials and Methods

### Isolation of Vegetative and Generative Nuclei

The vegetative and generative nuclei were isolated from lily pollens as described previously (Yang et al., 2016). In brief, mature pollen grains were collected from lily (*Lilium davidii* var. *unicolor*) at anthesis and stored at −80°C. The stored mature pollen grains were pre-hydrated, washed with 15% sucrose solution to remove lipid materials enclosing pollen grains, and then incubated in 15% sucrose solution at 27°C for 50 min to germinate. These just-germinated pollen grains were pooled using a 300-mesh hydrated screen, then osmotically shocked in pre-cooled isolation buffer (IB) (10 mM MES/KOH, pH 6.0, 5 mM EDTA, 10 mM NaCl, 0.15 mM spermine, 0.5 mM spermidine, 0.5 mM PMSF, 1 mM DDT, and 7.5% sucrose) for 5 min to release VNs and GCs. After removing cell debris using a 400-mesh screen, VNs were collected by centrifugation at 1,000 × *g* for 5 min and further purified using 1,500 × *g* for 20 min in a 7.5% Percoll solution. GCs were collected by centrifugation at 500 × *g* for 5 min, and further purified using 1,000 × *g* for 20 min in the 18%/24% Percoll solution. Purified GCs stayed at the interface of 18 and 24% Percoll, and then were collected with a dropper carefully. At last, GCs were treated with 0.1% Triton X-100 in IB at 4°C for 10 min, and the released GNs were collected by centrifugation at 1,000 × *g* for 5 min. Purified VNs and GNs were snap-frozen in liquid nitrogen and stored at −80°C.

### Morphological Observation of Vegetative and Generative Nuclei

The purified VNs and GNs were stained with aceto-carmine (1% fuchsin and 45% glacial acetic acid, with one drop of 4% FeSO_4_), and the morphological characteristics and the purity of nuclei were examined under a microscope (Axio Scope.A1, Zeiss).

### Nuclear Protein Extraction and Purity Assessment

The nuclei were broken by sonification in extraction buffer containing 0.15 M NaCl, 20 mM Tris–HCl, pH 8.0, 10 mM EDTA, 1 mM PMSF, 10 mM dithiothreitol, and 1 × cocktail (Roche). A one-tenth volume of 1% rapigest (Waters) was added, and the mixture was homogenized for 20 min at 4°C. After centrifugation at 12,000 × *g*, the supernatant’s proteins were transferred to a new centrifuge tube, and protein concentration was determined by the Bradford method (Bradford, 1976) using a DU640 UV-visible spectrophotometer (Beckman). The purities of extracted nuclear proteins were assessed by Western blot. The primary antibodies against nuclear histone H3 (Abcam, ab1791) and cytoplasmic UDPase (Abcam, ab154817) were used at a dilution of 1:5000.

### Peptides Labeled by iTRAQ

Each 50 μg of nuclear protein from VN and GN samples was digested with 10 ng/μl trypsin (Thermo Scientific) according to the FASP procedure described by Wisniewski et al. (2009). The resulting peptide mixture was labeled using the 4-plex iTRAQ reagent, according to the manufacturer’s instructions (AB Sciex). Peptides from VN were labeled with iTRAQ tags 114 and 115, respectively, and peptides from GN were labeled with iTRAQ tags 116 and 117, respectively. Subsequently, equal amounts of peptides labeled with iTRAQ tags 114 and 116 were pooled together, and peptides labeled with iTRAQ tags 115 and 117 were pooled together for a total of four repeats (Supplementary Figure 1). Each set of pooled peptides was dried using a vacuum drier for further high-pH reversed-phase chromatography fractionation.

### High-pH Reversed-Phase Chromatography Fractionation

After being reconstituted in 0.1% formic acid, the peptide mixtures were loaded onto a 4.6 mm × 250 mm Durashell-C18 column packed with 5-μm beads with a 300-Å pore (Agela) and fractionated by an Agilent 1290 Infinity UHPLC system. The mobile phases consisted of buffer A (20 mM ammonium formate, pH 10) and buffer B (80% acetonitrile in 20 mM ammonium formate, pH 10). The flow rate was maintained at 0.8 ml/min for 65 min and optimized eluting condition was as follows: linear binary gradient from 5 to 15% buffer B for 25 min, 15 to 38% buffer B for 15 min, 38 to 90% buffer B in 1 min, isocratic at 90% for 8.5 min, and returned to 5% buffer B for 10.5 min. The UV detector was set at 210 nm, and fractions were manually collected every minute and later pooled together. The elution fractions were collected and combined into six pools and then desalted and dried by a vacuum drier for subsequent MS identification.

### Protein Identification by Nano LC-MS/MS

After being reconstituted in 0.1% formic acid, each pool was analyzed using an Eksigent NanoLC Ultra 2D Plus platform coupled to a TripleTOF 5600^+^ mass spectrometer (AB Sciex). The peptides in each pool were first desalted on a 100-μm × 20-mm trap column and then separated on an analytical 75-μm × 150-mm column. Both columns were filled with Magic C18-AQ with particle diameter 5 μm and pore size 200 Å (Bruker Michrom Bioresources). The mobile phase A was 0.1% formic acid in ultrapure water, while mobile phase B was 0.1% formic acid in acetonitrile. Peptides were eluted in a linear gradient of 5–32% mobile phase B over 70 min at a 300 nl/min flow rate. Precursor ions were selected across the mass range of 350–1,500 m/z in high-resolution mode (>30,000) using 250 ms accumulation time per spectrum. A maximum of 25 precursors per cycle from each MS spectrum was selected for fragmentation with 100 ms minimum accumulation time for each precursor and dynamic exclusion for 20 s. Tandem mass spectra were recorded in high-sensitivity mode (resolution >15,000) with the options of rolling collision energy and iTRAQ reagent collision energy adjustment selected.

MS/MS spectra were searched using ProteinPilot^TM^ 4.5 software (AB Sciex) against the lily protein database (NCBI SRA:SRP066393) with the following parameters: (1) Sample Type: iTRAQ 4-plex (Peptide Labeled); (2) Cysteine Alkylation: MMTS; (3) Digestion: Trypsin; (4) Instrument: TripleTOF 5600; (5) Species: None; (6) Quantitate: Yes; (7) Bias Correction: Yes; (8) Background Correction: Yes; (9) Search Effort: Thorough; (10) FDR Analysis: Yes. For iTRAQ quantitation, the peptide for quantification was automatically selected by the Pro Group^TM^ algorithm to calculate the reported peak area. The quantitative ratio is supported by four repeats, and the *p* value of each quantitative ratio is below 0.01 (see Supplementary Table 2).

A reverse database search strategy was applied to estimate the global FDR for peptide identification. The differentially expressed proteins (DNPs) were further analyzed, which should meet the unused score greater than four and *p*-value less than 0.05.

### Bioinformatic Analysis

The DNPs were annotated via Arabidopsis homologs using TAIR BLAST 2.2.8 with the *E*-value less than 10^––3^. Proteins were functionally categorized according to the molecular and biological functions of the corresponding Arabidopsis homologs. For GO enrichment analysis, the annotated proteins were first submitted to PlantGSEA^1^ and then summarized and visualized by REduce VIsualize Gene Ontology (REVIGO^2^). The PPI network was predicted using the STRING version 11^3^, with the confidence edge greater than 0.7. The DNPs involved in the metabolic pathway were analyzed by the KAAS program^4^. For transcription factor prediction, these DNPs were blasted against Plant Transcription Factor Database^5^ and Arabidopsis thaliana transcription factor database^6^.

### Validation of Key Differentially Expressed Proteins by Western Blot

Nuclear proteins extracted from VNs and GNs were separated by 12% SDS-PAGE and then electrophoretically transferred onto PVDF membrane (Thermo Fisher) using a transfer buffer containing 25 mM Tris, 192 mM glycine, and 20% methanol, then immunodetected as described previously (Dai et al., 2007; Yang et al., 2016). The primary antibodies against AGL104 and SSRP1 were raised in rabbits using an antigenic determinant specific for the respective protein produced by B&M Biotech^7^, and an antibody of H4 was purchased from Merck Millipore.

### Phenotypic Analysis of Homologous Genes in Arabidopsis Mutants

*Arabidopsis thaliana* (Col-0), the T-DNA insertion *ssrp1* (SALK_001283) from Nottingham Arabidopsis Stock Centre (NASC^8^), and *agl104* (SALK_098698) mutant from AraShare^9^ were grown in soil in a phytochamber at 22°C under 16-h photoperiods. Polymerase chain reaction (PCR)-based genotyping of the plant lines was applied to screen the homozygous mutants. For analysis of seed setting rate, siliques from the mutant and wild lines were collected and opened with sharp forceps to count the seeds. For morphological characterization of pollen grains, the mature pollen grains were collected into a DAPI staining solution (0.1 M sodium phosphate, pH 7.0, 1 mM EDTA, 0.1% Triton X-100, and 0.25 mg ml^–1^ DAPI). After incubation for 15 min, the stained pollen grains were examined under UV light using a fluorescence microscope. The *in vitro* germination and pollen tube growth rate assay of the pollen grains was performed in a liquid pollen growth medium (1 mM KCl, 10 mM CaCl_2_, 0.8 mM MgSO_4_, 0.01% H_3_BO_3_, and 18% sucrose) at 22–24°C for 3 h.

## Results

### Isolation and Morphological Observation of VNs and GNs

**FIGURE 1:**
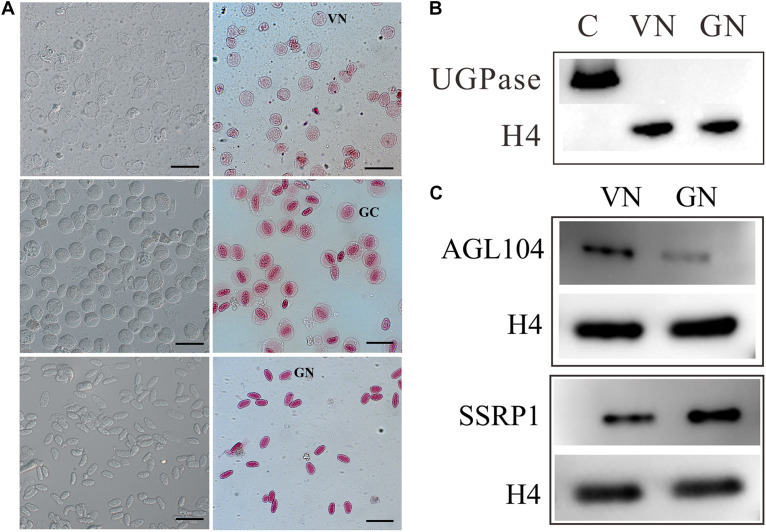
Preparation of nuclear proteins from purified vegetative cell nucleus, VN, and generative cell nucleus, GN. **(A)** Cytological characterization of purified VC nuclei (VN) and GC nuclei (GN) isolated from lily (*Lilium davidii*) pollens. The images of isolated VNs, GCs, and GNs with aceto-carmine staining were acquired and displayed from the top to bottom row. Scale bars = 100 μm. **(B)** Western blot examination of nuclear protein purity. Proteins extracting from VN and GN were separated by 12% SDS-PAGE, transferred to PVDF membranes, and detected with antibodies for UGPase, a cytoplasmic marker, and H4, a nucleus marker. **(C)** Western blot analysis of the expression patterns of AGL104 (Unigene5688) and SSRP1 (CL774.Contig1) in VN and GN.

To gain insight into the pollen cell fate differentiation mechanism, we compared the nuclear proteome profiles of VC and GC. VNs were isolated from just germinated pollen using 7% Percoll density centrifugation, while GNs were acquired from purified GCs, which are released from bursting of VC and then purified using 18%/24% Percoll density centrifugation. The isolated VNs and GNs are with high purity and without cross-contamination (Figure 1A). VNs are almost spherical (20–30 μm in diameter) and showed light red by aceto-carmine staining (Figure 1A), while the isolated GCs are spherical (35–40 μm in diameter) and GNs have an ellipsoid shape (long axis of approximately 30 μm) with deep aceto-carmine staining (Figure 1A). In accordance with the previous study, the nuclear staining results confirmed that VNs’ chromatin is less condensed than that of GNs.

### Identification of DAPs Between VN and GN

**FIGURE 2:**
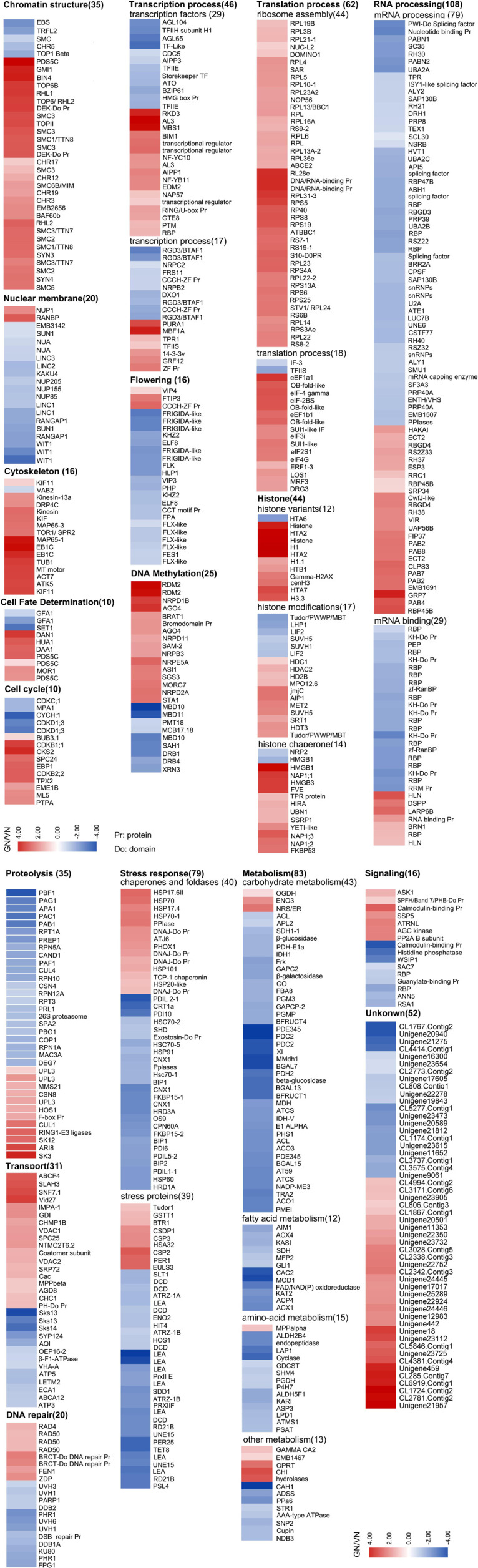
Abundance patterns of 720 DAPs revealed from the proteomic analysis. The different “ribbons” represent various functional categories with functional annotation and protein number at the top of each “ribbon.” The rows of the “ribbon” represent individual protein, and the abbreviation for each protein and accession numbers of unknown proteins are listed on the right side (see detailed protein information in Supplementary Tables 2, 3). The scale bar indicates log (base2)-relative protein abundance ratios between GN and VN. Protein highly expressed in VN or GN was indicated in red or blue, respectively.

The purity of nuclear protein extracted from VNs and GNs was evaluated by the Western blot (Figure 1B). The signal of Histone 4 (H4, as a nuclear marker) was detected in both VN and GN samples, while Uridine 5′-diphosphatase (UDPase, as a cytoplasmic marker) was not detected in any of them. This result indicated that nuclear protein was prepared without cytoplasmic protein contamination. Nuclear proteins were subsequently quantified and identified using the iTRAQ-based quantitative proteomic method. Four experimental repeats were conducted. To identify more DAPs, labeled peptides were firstly separated by two-dimensional liquid chromatography and then identified by tandem mass spectrometry. After separation by high-pH reversed-phase chromatography, each minute of effluent was collected and combined into the six fractions to reduce sample complexity (Supplementary Figure 2). Each fraction was re-separated and identified by nano LC-MS/MS. When searching the custom-made lily protein database and applying the rigorous identification criteria [1% false discovery rate (FDR), unused score ≥ 4], about 2,000 nuclear proteins were identified (Supplementary Table 1). Among them, 720 nuclear proteins are differentially expressed between VNs and GNs with expression change level ≥1.5 or ≤0.67 (Figure 2 and Supplementary Tables 2, 3). The DAPs were divided into two groups: 379 DAPs highly expressed in VNs and 341 DAPs highly expressed in GNs. These results implied that numerous proteins might be involved in the VC and GC fate differentiation.

### Functional Categories of Differentially Expressed Nuclear Proteins

**FIGURE 3:**
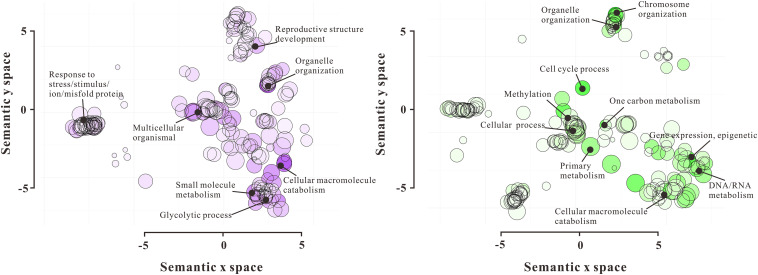
GO functional enrichment analysis of 720 DAPs by REVIGO. The left scatterplot shows significantly enriched GO terms related to biological processes in VN, and the right scatterplot showed significantly enriched GO terms in GN. GO terms are represented by circles and are clustered according to semantic similarities to other GO terms in the gene ontology (more general terms are represented by larger size circles, and adjoining circles are most closely related). Bubble color indicates the log10P-value for the enrichment derived from the AgriGO analysis, and bubble size is proportional to the frequency of the GO term. The statistically most significant GO terms are highlighted by annotation.

To gain the functional pieces of information of 720 DAPs, these proteins were functionally annotated by their homologs after searching against NCBI non-redundant and TAIR databases using the Basic Local Alignment Search Tool (BLAST). According to their molecular function, the 720 DAPs were divided into 18 categories, including Chromatin Structure (35), Nuclear Membrane Structure (20), Transcription Process (46), Translation Process (62), RNA Processing (108), Cytoskeleton (16), Cell Fate Determination (10), Flowering (16), DNA Methylation (25), Histone (44), Cell Cycle (10), Signaling (16), Transportation (31), Stress Response (79), Ubiquitin-related Proteolysis (35), DNA Repair (20), Metabolism (83), and Function Unknown (52) (Figure 2 and Supplementary Table 3). Furthermore, GO enrichment analysis by REVIGO showed a significant molecular function difference between VN and GN (Figure 3). In VN, DAPs are mainly involved in Reproductive Structure Development, Organelle (nuclear envelope) Organization, Stress Response, and Metabolism, while in GN, DAPs mainly participated in biological processes including Chromosome Organization, Cell Cycle, Methylation, Epigenetic Regulation, and Metabolism. Collectively, these results illustrated that VC and GC have evident functional divergence.

### Identification of Transcription Factors and Cell Fate Determinants

Transcriptional regulation is a particularly important aspect of cell fate differentiation. There are 46 DAPs involved in the transcription process. Among them, 29 proteins were verified as TFs and assigned to 13 TF families, including MYB (1), ARID (1), NAC (1), GeBP (4), C2H2 (4), PHD (1), NF-YB (1), CCAAT-HAP5 (1), RKD (1), Alfin-like (2), MADS (2), bZIP (1), and bHLH (1). Among them, 12 TFs were highly expressed in VNs, while 17 TFs were highly expressed in GNs (Figure 2 and Supplementary Table 4). The TFs highly expressed in VN were mainly involved in the regulation of gametic cell fate (ATO) and pollen tube development (AGL65, AGL104, BZIP61, and HMG box protein with ARID domain), cell cycle (CDC5), and RNA processing (AIPP3). The TFs highly expressed in GN participated in the regulation of cell differentiation (RKD3, AL3), male fertility (BIM1), male gametogenesis (NF-YB11, NF-YC10), and RNA processing (NAP57, AIPP1, and EDM2). More notably, three functionally unknown DNA-binding storekeeper protein-related transcriptional regulators were highly expressed in GNs. In addition to TFs, 10 DAPs may have a role in cell fate determination, such as DUO1-activated ATPase 1 (DAA1), D Nuclduo1-Activateeic Acid Binding Protein 1 (DAN1), GEM1, and Precocious Dissociation of Sisters 5 (PDS5C) (Figure 2 and Supplementary Table 3).

### Analysis of the DAPs Interaction Network by String

**FIGURE 4:**
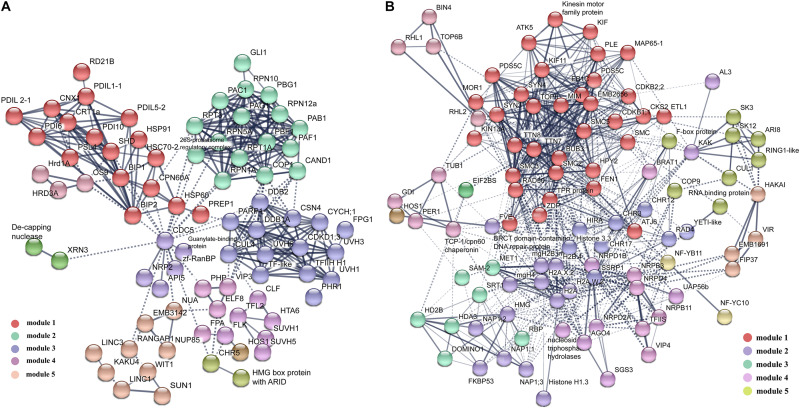
The protein–protein interaction (PPI) network in VN and GN revealed by STRING analysis. **(A)** Among 379 DAPs highly expressed in VN, 88 proteins were analyzed, except proteins involved in metabolism and RNA processing. **(B)** Among 341 DAPs highly expressed DAPs in GN, a total of 101 proteins were analyzed, except proteins involved in ribosome assembly and translation processes. The networks were generated with protein interactions score > 0.7 and MCL inflation parameter 3. Different modules were indicated in different colors. For the PPI networks of excluded proteins, see Supplementary Figures.

To reveal how these DAPs synergically regulated cell fate differentiation, the two groups of DAPs were analyzed using the protein interaction network analysis tool. For DAPs highly expressed in VN, 60 proteins form one RNA processing module (Supplementary Figure 3A), and 57 proteins form a metabolic interaction network (Supplementary Figure 4). The rest of the 88 DAPs formed a complex interaction network with five main functional modules, including Misfolded Proteins Response, Ubiquitin-Related Proteolysis, DNA-Damage Repair, Floral Organ Development, and Nuclear Membrane Organization (Figure 4). For DAPs highly expressed in GN, 51 proteins formed a functional module involved in Ribosome Assembly and Translation (Supplementary Figure 3B), the rest of 101 DAPs formed an interaction network with five main functional modules including Chromatin Remodeling and Cell Cycle, Histone Variants and Chaperones, Histone Modification, DNA Methylation, and Ubiquitination (Figure 4).

### KEGG Pathway Analysis

**FIGURE 5:**
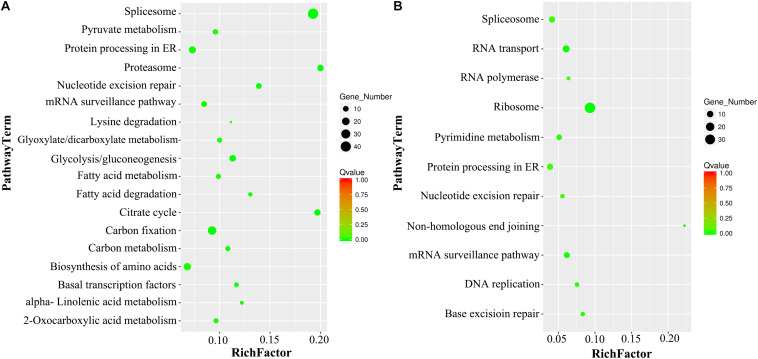
KEGG pathway enrichment analysis of DAPs in VN and GN. **(A)** The scatter diagram of the top 18 statistics from KEGG pathway enrichment in VN. **(B)** The scatter diagram of the top 11 statistics from KEGG pathway enrichment in GN. The *Q*-value must be <0.05, and the Rich factor positively correlates with the degree of enrichment.

After the KEGG analysis, the most significantly enriched VN metabolism pathways were Spliceosome, Citrate Cycle, Proteasome, Carbon Metabolism, Glycolysis, Biosynthesis of Amino Acids, and Fatty Acid Metabolism, while the GNs were enriched in Ribosome, RNA Transport, mRNA Surveillance pathway, DNA Replication and Repair, Protein Processing in ER, and Nucleotide Metabolism (Figure 5).

### Verification of DAPs Expression by Western Blot

The DAPs were carefully screened, and the differential expression levels of each DAP were supported by four replicates of mass spectrometry data. To further validate the expression patterns of proteins detected by the iTRAQ proteomic approach, some DAPs were selected and examined by Western blot analysis. MIKC^∗^-type (MADS DNA-binding domain, Intervening domain, Keratin-like domain, and C-terminal domain) factors, a divergent clade of MADS box genes, are enriched in mature pollen and may play important regulatory roles in pollen tube development. In our data, AGAMOUS-LIKE104 (AGL104) belongs to this clade and is highly expressed in VN. Moreover, histone chaperones play critical roles in cell fate determination that mediate nucleosome disassembly and reassembly. Structure-specific recognition protein 1 (SSRP1), a subunit of the histone chaperone, facilitates chromatin transcription (FACT) complex. It not only acts as one essential histone chaperone but also directly associates with elongating RNA polymerase II (RNAPII) along the transcribed region of genes. Despite the intense research on animals, little has been done on plants, let alone on the differentiation of pollen cell fate. In our data, SSRP1 was identified and highly expressed in GN. So we chose these two DAPs with great research significance for Western blot analysis. The Western blot results show that the expression level of AGL104 was higher in VN than in GN, while the expression of SSRP1 was lower in VN than in GN (Figure 1C). The Western blot results of these two proteins are consistent with the MS results that indicated the reliability of the proteomic quantitation.

### Analysis of DAPs Functions in Pollen Development Using the Homologous Gene Mutants in Arabidopsis

**FIGURE 6:**
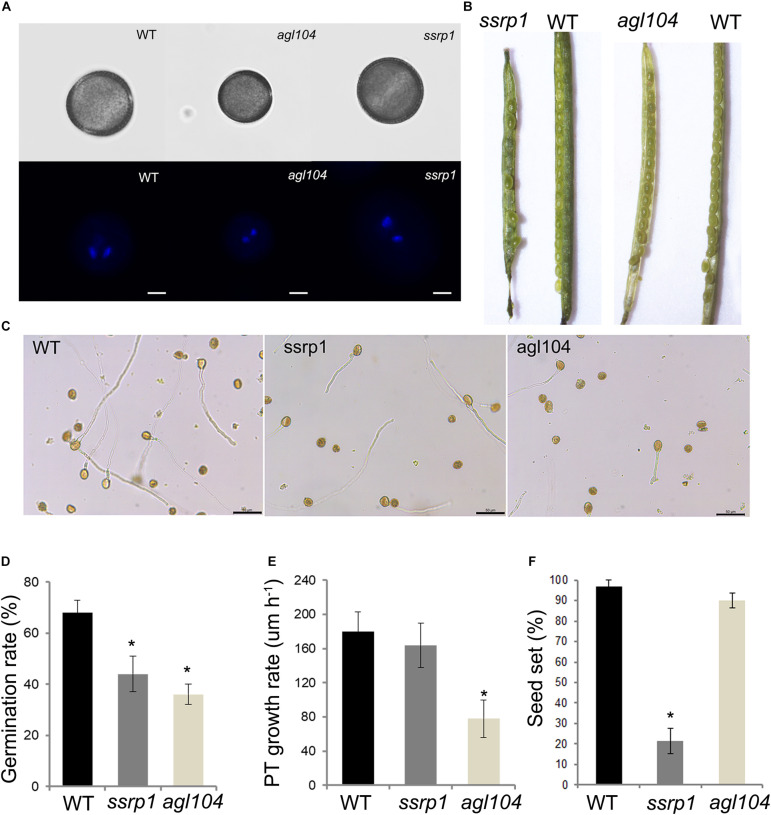
Phenotypic characterizations of the homologous gene mutants in Arabidopsis. **(A)** Fluorescent microscopy of the pollens stained with DAPI, Bars = 20 μm. **(B)** The seed setting in silique shows the reduced seeds of *ssrp1* mutant. **(C)** Pollen grains germinated in a liquid pollen growth medium for 3 h, showing the reduced pollen germination rate of *ssrp1 and agl104* mutants. Bars = 50 μm. **(D)** *In vitro* pollen germination rate assay. The results are presented as mean ± s.e. **(E)** Pollen tube growth rate assay. The difference in length between the two-time points was divided by the time duration to yield the average pollen tube growth rate (expressed in μm h^– 1^). The results are presented as mean ± s.e. **(F)** The seed setting assay. The results are presented as mean ± s.e.; * statistically significant differences with *p* < 0.05 by Student’s *t*-test.

To investigate further the function of these two DAPs SSRP1 and AGL104, the phenotypes of homologous gene mutants in Arabidopsis were explored in detail. Similar to the Col-0, both *Atssrp1* and *Atagl104* mutants could form the typical mature pollen with one vegetative nucleus and two sperm nuclei (Figure 6A). Impressively, compared to the fully elongated Col-0 and *Atagl104* mutant siliques, the majority of *Atssrp1* mutant siliques were partially elongated and only contained four to seven seeds (Figure 6B). This observation is supported by the statistical analysis results presented in Figure 6F. Next, the *in vitro* pollen germination assay results revealed that both mutants have lower pollen germination rates than Col-0, especially only 36% in the *Atagl104* mutant (Figures 6C,D). Besides, the *Atagl104* mutant pollen tube grew more slowly than did those of the wild type (Figure 6E). These results indicate that SSRP1 and AGL104 may significantly impact lily pollen cell differentiation and highlight our proteomic data’s significance.

## Discussion

In flowering plants, asymmetric division of the haploid microspore produces two daughter cells with distinct fates: the large VC quit cell cycle with 1C DNA content protrudes a pollen tube to deliver the two SCs; the diminutive GC with 2C content enclosed in the cytoplasm of VC undergoes further mitosis to generate SCs. Many studies suggest that asymmetric division plays an essential role in cell fate determination, but limited knowledge is available about the differential molecular landscapes between VC and GC after differentiation (Gallagher and Smith, 1997; Chaturvedi et al., 2016).

**FIGURE 7:**
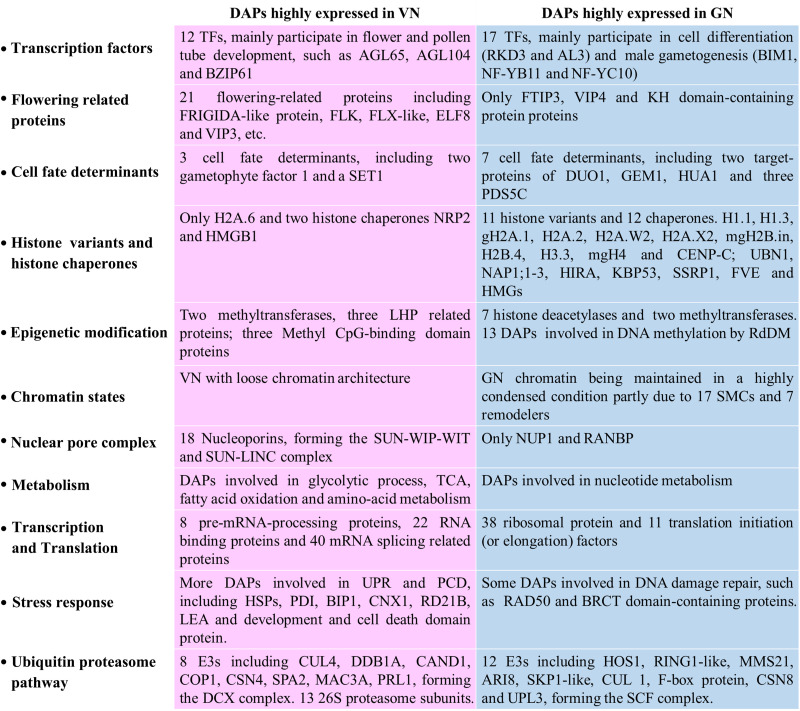
Summary of the prominent protein composition difference between VN and GN after fate differentiation.

Our previous study revealed that differential histone programs are established following the asymmetric division and important for identity establishment and differentiation of pollen cells (Yang et al., 2016). Moreover, Okada et al. (2007) identified 356 gene transcripts that were enriched in lily GCs, including some ribosomal protein, histone deacetylase, elongation factor, ubiquitin-pathway-related proteins, and five transcription factors, most of which were also identified in our study (Supplementary Tables 2, 3). Consistent with our finding, Liu et al.’s (2018) transcriptome work showed that the featured GO terms in SC lineage for cell cycle, DNA metabolic process and DNA methylation, chromatin-related pathways, and cell differentiation differed from those in the VC (or mature pollen grain), which largely involved polar pollen tube growth. In this study, we comprehensively compared the nuclear proteome of VC and GC and further analyzed the functions of 720 DAPs that give a differential protein landscape between VC and GC for the first time (Figure 7).

### TFs in VN Are Involved in Pollen Tube Development, While TFs in GN Are Involved in Cell Differentiation

Transcription factors are key components regulating the cell fate differentiation, while the overall TFs profile of VCs and GCs have not been addressed. In this work, 29 DAPs were verified as TFs, and some of them were revealed for the first time. Among the 12 TFs highly accumulated in VNs, CDC5 (Unigene22395) is an MYB-related TF. AtCDC5 was reported to regulate the G2/M phase transition of the cell cycle in shoot apical meristem (Lin et al., 2007). Considering the VN staying at the G1 phase, we speculated that CDC5 is more likely playing another role in VN. Recently, some studies suggested that CDC5 can regulate the accumulation of microRNAs (miRNAs) by forming MOS4-associated complex with MAC3A and PRL (Zhang et al., 2013; He et al., 2015; Li et al., 2018). Not surprisingly, MAC3A (Unigene16511) and PRL1 (Unigene24560) are also highly accumulated in VNs, implying that CDC5 has evolved a non-canonical mechanism to regulate gene expressions in VNs.

In Arabidopsis, AGL66 and AGL104 belong to the pollen-specific MIKC^∗^ MADS box proteins and are important for pollen growth (Verelst et al., 2006, 2007; Adamczyk and Fernandez, 2009). Besides, Xia et al.’s (2014) paper showed that AtHMGB15 functions in pollen tube growth through interaction with the AGL66 and AGL104. Our proteomic data support these previous observations, on account of that AGL66 (Unigene16864), AGL104 (Unigene5688), as well as one TF with HMG box (CL5179.Contig2) are all highly expressed in VNs. Moreover, our results further confirm that *agl104* single mutant in Arabidopsis reduces the pollen germination and delays pollen tube growth (Figures 6C–E).

It has been well established that bZIP TFs have critical roles in plants. Similar to the expressions of AtbZIP61/34/18 in pollen and regulation pollen development (Gibalova et al., 2009, 2017), we also found a bZIP61 (Unigene16424) highly expressed in VNs. In female gametic cell fate specification, several lines of evidence suggested that gametic cell fate restriction is specifically coupled to the function of various core spliceosomal components, including ATO (Moll et al., 2008). Interestingly, the ATO (CL1029.Contig2) and other spliceosomal components were also found in VNs, and whether ATO has a similar regulatory function in male cell fate differentiation needs further study.

We also identified 17 TFs, which are highly expressed in GN. Some of them attracted our attention. RKD3 (Unigene24902) belongs to the plant-specific RWP-RK transcription factors, and the protein amounts of RKD3 in GNs are 18.5-fold higher than that in VNs. Consistent with our data, RWP-RK proteins were also found in the sperm nuclei of *Pleodorina starrii* and *Marchantia polymorpha*, suggesting functioning in male gametogenesis (Nozaki et al., 2006; Koi et al., 2016). Interestingly, RKDs were also expressed in female gametes and verified to be involved in the differentiation of female gametes in Arabidopsis and *M. polymorpha* (Chardin et al., 2014; Tedeschi et al., 2017; Hisanaga et al., 2019).

Moreover, AL3 was also identified as a regulator of cell differentiation during gametophyte development in *Physcomitrella patens* (Xiao et al., 2011). In our data, the protein amounts of AL3 (CL7121.Contig1) in GNs are 31.5-fold higher than that in VNs. Recent studies showed the VIIIa bHLH proteins as core and conserved regulators for reproductive development, including germ cell differentiation (Yamaoka et al., 2018). In our data, BIM1 (CL4663.Contig1) belongs to bHLH and accumulates in GNs. In Arabidopsis, BIM1 is involved in male gamete development, acting together with Squamosa Promoter Binding Protein-Like 8 (SPL8) (Chandler et al., 2009; Xing et al., 2013). Therefore, it would be of significance to further study the functions of BIM1 in lily pollen.

MBS1 (CL131.Contig1) and PTM (Unigene25241) were also highly expressed in GNs, while the role of these two TFs in GNs are still unclear. The conserved TF NF-Y is a heterotrimer and comprises NF-YA, NF-YB, and NF-YC. In Arabidopsis, NF-YA1/5/6/9 accumulates in pollen and plays redundant roles in male gametogenesis (Mu et al., 2013). In our data, NF-YB11 (Unigene5682) and NF-YC10 (CL4468.Contig1) are highly expressed in GNs. To ensure the genetic information’s stability, epigenetic silencing marks, such as DNA methylation and H3K9me2, are enriched in the genomes of GNs, while some heterochromatin-containing genes may be vital for GC specification. How is the expression of these genes regulated? Recently, an elegant study indicated that a complex contains ASI1-Immunoprecipitated Protein 1 (AIPP1), Enhanced downy mildew 2 (EDM2), and Anti-silencing 1 (ASI1) function in promoting the expression of heterochromatin-containing genes (Duan et al., 2017). However, the function of AIPP3 is antagonistic to the AIPP1–EDM2–ASI1 complex (Duan et al., 2017). Remarkably, in our data, AIPP1 (CL900.Contig3), EDM2 (Unigene24317), and ASI1 (CL3185.Contig5) are highly expressed in GNs, while AIPP3 (CL900.Contig3) are highly expressed in VNs.

The R2R3 MYB-type transcription factor DUO1 was the first TFs required for the differentiation of male gametes. In this study, we identified two important cell fate determinants acting downstream of DUO1, namely, DAA1 (Unigene21815) and DAN1 (CL4279.Contig1). These two determinants were also identified by other researchers in Arabidopsis and lily GCs (Okada et al., 2006a; Wang et al., 2008). Twell et al. (2002) showed that GEM1, a microtubule-associated protein, is required for male gamete specification. Our data also identified a GEM1 (Unigene23904) highly expressed in GNs and confirmed that earlier observation. Moreover, three PDS5C (Unigene23096, CL4067.Contig2, and Unigene25058) were highly expressed in GNs, and their homologs in Arabidopsis were reported to have a role in the meiotic division, DNA repair, and germ cell development (Pradillo et al., 2015).

### Differential Histone Programs Between VN and GN Mediated by Specific Histone Chaperones

Many studies indicated that diverse histone variants would differentially express between VCs and male germline cells to specify the cell differentiation by affecting gene expression. In Arabidopsis and lily, different H3 variants have been assembled into the chromatin of VC and germ cells (Xu et al., 1999; Okada et al., 2005; Ingouff et al., 2007).

In this study, we identified 12 histone variants specifically accumulated in GN of lily pollen as in our previous study (Yang et al., 2016). More importantly, our data provide important clues as to how these differential histone patterns were established. Specific histone chaperones are essential for histone variants incorporating at specific locations and the dynamic nature of chromatin. In our study, 14 histone chaperones were identified, and 12 of the 14 chaperones are highly expressed in GNs. These histone chaperones include H2A-H2B chaperones Nucleosome assembly protein1;1 (NAP1;1, CL6632.Contig2), NAP1;2 (CL5457.Contig2) and NAP1;3 (Unigene23868) (Mazurkiewicz et al., 2006; Chen et al., 2016), H3.3-specific chaperone Ubinuclein 1 (UBN1, Unigene1409) and Histone regulator A (HIRA, Unigene25726) (Ricketts et al., 2015), H3-H4 histone chaperone FK506-binding protein 53 (FKBP53, Unigene22477), and H1 histone chaperone structure-specific recognition protein 1 (SSRP1, CL774.Contig1) (Pfab et al., 2018; Falbo et al., 2020; Singh et al., 2020).

In Arabidopsis, as the histone chaperone’s subunit facilitates chromatin transcription (FACT) complex, SSRP1 has a role in maintaining cell identities and pollen fertility (Hossan et al., 2016; Frost et al., 2018; Kolundzic et al., 2018; Pfab et al., 2018). Our results further confirm that depletion of AtSSRP1 proteins has normal phenotypic mature pollen but leads to severe effects on the seed set (Figures 6A,B,F). Some studies reveal that AtSSRP1 is required for DNA demethylation and activation and repression of many parentally imprinted genes in the central cell. Whether AtSSRP1 could influence the chromatin state of GN is a very intriguing question. The remaining two chaperones, NAP1-related protein 2 (NRP2, Unigene20745) and High mobility group B1 (HMGB1, Unigene26501), are highly expressed in VNs, which may be responsible for histone variant assembly into the chromatin of VNs. Therefore, we conclude that the differentially expressed histone chaperone leads to the differential histone patterns. Investigating the functions of these chaperones in pollen would be a vital direction to understand the mechanism of pollen cell fate differentiation.

### Highly Condensed Chromatin in GN Partially Maintained by Some SMC and Chromatin Remodelers

One of the significant differences in nuclear morphology between VNs and GNs is the chromatin state. GN has a more condensed chromatin organization. Besides the distinct histone variants and modifications that have dynamic and differential expressions in VNs and GNs, our data also show that more SMCs and chromatin remodelers are expressed in GNs, such as SMC1/3 (Unigene18130 and Unigene21503), Sister chromatid cohesion 1 protein 3 (SYN3 and Unigene25603), SMC2 (Unigene20902), and SMC5/6 (Unigene23407 and Unigene16790) (Supplementary Table 3). It has been well established that SMC1/3 and SYN3 are important components of the cohesion complex, and SMC2/4 and SMC5/6 can form the condensin complex. Both cohesion and condensin complexes are essential for proper chromosome segregation during the mitosis of the germ cells. Similarly, SMC1/3 and SYN1 load sequentially in tomato microsporocytes (Qiao et al., 2011). SMC2/4 was reported to express in meristematic cells preferentially, and disruption of the Arabidopsis *SMC2/4* genes compromises male and female gametogenesis (Siddiqui et al., 2003, 2006). SMC6 is highly expressed in SCs and essential for spermatogonial differentiation (Verver et al., 2013).

Chromatin remodelers could further compact the chromatin into the higher-order structure. In this study, we identified some chromatin remodeling factors, such as SWI/SNF chromatin remodeling factor BAF60b (CL1705.Contig1) and DEK domain-containing proteins (Unigene15562 and Unigene22480), which are highly expressed in GNs. In animals, SWI/SNF chromatin remodeling complexes are known to modulate chromatin accessibility and essential for spermatogenesis (Menon et al., 2019). The evolutionarily conserved DEK domain containing proteins are implicated in critical chromatin-related processes. In Arabidopsis, AtDEK3 can affect nucleosome occupancy and chromatin accessibility and modulate the gene expression (Waidmann et al., 2014).

### The Morphological Differences of Nuclear Membranes Between VNs and GNs May Affect the Differentiation of Cell Fate

The nuclear envelope (NE) separates the cytoplasm from the nucleoplasm by a double membrane system and is perforated by nuclear pore complexes (NPCs), which are large multi-protein channels mediating macromolecular import and export (Capelson et al., 2010; Grossman et al., 2012). Among the 20 DAPs involved in NE structure, 18 DAPs are highly expressed in VNs. This result implies that more NPCs occurred in the NE of VNs than that of GNs. A similar phenomenon was also detected in NE of tobacco BY-2 cells that the dividing and quiescent cell nuclei harbor different proportions and conformations of NPCs (Fiserova et al., 2009).

In our study, nucleoporins were found, mainly including the inner-ring nucleoporins NUP155 (Unigene20026) and NUP205 (Unigene21043 and Unigene9598), outer-ring nucleoporin NUP85 (CL3210.Contig1), and two NUA (Unigene21057 and Unigene24368) presented in inner basket filament (Grossman et al., 2012; Zhu et al., 2017). Remarkably, we also identified one important NE protein, SAD1/UNC-84 domain protein 1 (SUN1 and Unigene24069), which can interact with other identified nucleoporins, such as WPP domain-interacting tail-anchored protein 1 (WIT1, Unigene19650, CL7050.Contig2, and Unigene19653), Little Nuclei 1 (LINC1, CL820.Contig1, and CL820.Contig2), LINC2 (Unigene23646), LINC3 (Unigene23645), RanGAP1 (Unigene18651 and Unigene18652), and KAKU4 (CL5772.Contig1) (Figure 4A).

Cumulative evidence showed that SUN1 and SUN2 are inner nuclear membrane localization and could form the linker of the LINC (nucleoskeleton and cytoskeleton) complex, which is a structure that spans the NE to link the nucleoskeleton and cytoskeleton (Crisp et al., 2006; Kim et al., 2015). Consistent with our data, AtSUN1/2 can interact with WITs forming the SUN–WITs complex, which is essential for pollen vegetative nuclear migration and successful pollen tube reception (Zhou and Meier, 2014). Similarly, a recent study also showed that AtKAKU4 protein was highly abundant on the envelopes of VN and less abundant on the envelopes of SC nuclei. In this study, the authors found that *KAKU4* deficiency affected the VN nuclear morphology and the migration order of the VN nucleus and SCs in pollen tubes (Goto et al., 2020). Moreover, AtSUN1/2-AtWIP-AtWIT-AtKAKU1/4 could form a complex that has a role in mediating the nuclear shape and nuclear migration (Oda and Fukuda, 2011; Zhou et al., 2012; Tamura et al., 2013; Goto et al., 2014; Talamas and Capelson, 2015). Our data collectively reveal the morphological differences of nuclear membranes between VNs and GNs. This finding is meaningful because more and more studies unveil that highly dynamic and coordinated interactions between the genome and the NE may contribute to tissue-specific gene expression programs to determine cell fate (Talamas and Capelson, 2015; Gu, 2018; Gomar-Alba and Mendoza, 2019). In the future, further functional research about these NE-related DAPs may improve our understanding of cell fate differentiation mechanisms in pollen.

### Different Metabolisms and Transcriptional and Translational Activities in VN and GN

The KEGG metabolic pathway analysis results clearly show that VN and GN have a noticeable difference in the metabolic pathway. In VNs, more DAPs are involved in carbon and fatty acid metabolism for nourishing GCs and pollen tube development. Previous ultrastructural observations are consistent with our results. They found that starch grains and lipid droplets increased in Arabidopsis VCs during the GCs formation (Kuang and Musgrave, 1996). Moreover, we have also noticed that many DAPs are involved in mRNA transcription and processing. These DAPs contain a number of DNA-dependent RNA polymerases II (Pols II), splicing factors, UBP1-associated proteins, small nuclear ribonucleoproteins (snRNPs), DEAD-box RNA helicases, and RNA-binding proteins (Supplementary Figure 3A).

In GNs, feature metabolic pathways are translation and nucleic acid-related metabolism. Many DAPs are ribosomal proteins and translation initiation factors (Supplementary Figure 3B and Supplementary Table 3). The discrepant activities of transcription and translation between VNs and GNs are consistent with previous findings that some genes are specifically transcribed in the VC but predominantly translated in the male germ cells, such as *ABA-hypersensitive germination3* (AHG3) (Jiang et al., 2015). Besides, some histone deacetylase (HD2B, HDT3, and HDC1) and some proteins involved in small RNA pathways, such as the components of Pols IV/V (RDM2, NRPD1B, and NRPE5A), Argonaute 4 (AGO4), and Suppressor of gene silencing 3 (SGS3), are also highly expressed in GNs (see review by He et al., 2015, for details about small RNA in pollen). These results collectively suggest that GCs have lower transcriptional activity mainly due to gene silencing by RdDM and histone deacetylation.

### Activation of the UPR in VNs, While DNA Damage Repairs in GNs

Pollen is particularly sensitive to environmental conditions, which could disturb the protein homeostasis and modify the genome. In VC, pollen tube growth requires a highly active membrane trafficking system with an extensive endoplasmic reticulum (ER) (Deng et al., 2013). These demands elicit the UPR, which helps mitigate stress damage and supports pollen tube growth (Fragkostefanakis et al., 2016). Our data reveal that more heat shock proteins (HSP/DNAJ), ER luminal binding proteins (BiPs), and protein disulfide isomerases (PDIs) are expressed in VNs, which are responsible for protein folding in the ER.

In Arabidopsis, BiPs and SHEPHERD are essential for male gametophyte development and fertilization (Ishiguro et al., 2002; Maruyama et al., 2014). Some calnexins and calreticulins are also highly expressed in VNs, which are essential for secretory protein processing. Members of ER-associated degradation (ERAD) pathway and programmed cell death (PCD) are also induced in VNs, such as HRD1A (Unigene21483), HRD3A (Unigene24163), 26S proteasome subunits, development, and cell death domain proteins (Supplementary Table 3). Compared with VNs, more DNA repair proteins are expressed in GNs, such as RAD50, Flap endonuclease 1 (FEN1, Unigene20835), and BRCT-domain-containing DNA repair proteins (Unigene25473 and Unigene25474). Similarly, Xu et al. (1998) showed that DNA repair gene ERCC1 is upregulated in lily GCs. The DNA damage response pathways are important for the GN’s genomic integrity and stability to transmit accurate genomic information to the next generation. Moreover, VN and GN have very different ubiquitin degradation pathways. In GN, two SKP1-like proteins (CL1941.Contig1, CL1620.Contig1), one Cul1 protein (CL3643.Contig3), and one F-box protein (CL409.Contig9) could interact with each other and form SCF complex (Figure 4B). In Arabidopsis, the SCF^(FBL17)^ complex is essential for male germ cell proliferation and twin SC production by regulating the cell cycles (Kim et al., 2008). Another E3 highly expressed in GN is MMS21 (Unigene24076), which might require normal meiosis and gametophyte development as AtMMS21 (Liu et al., 2014). In VN, the highly expressed E3 is Cul4 (CL60.Contig1). The protein interaction results show that Cul4 interacts with DDB1 (Unigene22360), which could form a complex DCX (Figure 4A). Some pieces of evidence indicate that DCX is one of the key regulators in plant development and physiology (Zhang et al., 2008). DCX can regulate flowering time by interacting with VIP3 (Unigene8589) and ELF8 (Unigene23186 and Unigene23025) (Lee et al., 2008) or with LHP1 (CL1125.Contig1) (Pazhouhandeh et al., 2011). Collectively, these results suggest different stress response mechanisms and ubiquitin degradation pathways are reinforced in VC and GC.

## Conclusion

This study hypothesized that by systematically comparing the nucleoproteome differences between the VN and GN, more indications of cell fate differentiation could be found, such as new transcription and cell fate determinants, clarifying the reasons for differences in histone programs and different cell cycle regulation mechanisms, and unknown differences in molecular activities. The study results proved our hypothesis by providing a comprehensive nuclear protein comparison between VC and GC nuclei in lily using state-of-the-art quantitative proteomic techniques. The main results and conclusions of this study could be summarized as follows: (1) The nuclear proteomic analysis is currently the only method to uncover the systemic differences between VC and GC. (2) In this study, we have successfully isolated a large amount of VNs and GNs with high purity from lily pollens. (3) We identified 720 DAPs and grouped the results in 18 functional (Supplementary Table 3) and 11 fate differentiation categories (Figure 7). Among them, we identified 29 transcription factors (TFs) and 10 cell fate determinants. The identified novel transcription factors may play an important role in cell fate differentiation. (4) Significant differences were found in the molecular activities of vegetative and reproductive nuclei with respect to transcription factors, cell fate determination, histone variants and histone chaperons, epigenetic modifications, chromatin states, nuclear pore complexes, metabolism, transcription and translation, stress response, and ubiquitin proteasome pathway. In VN, DAPs are mainly involved in Reproductive Structure Development, Organelle (NE) Organization, Stress Response, and Metabolism. While in GN, DAPs mainly participated in biological processes including Chromosome Organization, Cell Cycle, Methylation, Epigenetic Regulation, and Metabolism. (5) The comprehensive analysis of the differences between the histone chaperones of VCs and reproductive nuclei allowed us to unravel the reasons for the differences in histone programming between the two. (6) The discrepant activities of transcription and translation between VNs and GNs are consistent with previous findings that some genes are specifically transcribed in the VC but predominantly translated in the male germ cells.

### Suggestions for Future Research Directions

The results of our study provide suggestions for several possible future research directions: (1) How do these histone chaperones regulate the dynamics of histone variants and thus affect cell fate determination. (2) The differences in the nuclear membranes between VCs and germ cells may also influence the differentiation of cell fate, which is a new direction for future research. (3) Moreover, proteins belonging to UPR signaling pathways are up-regulated in VN, while DNA damage repair proteins are highly represented in GN. This is an intriguing finding at the proteome level that could be further investigated in the future. (4) The different ubiquitin pathways identified by our research may be one of the factors that affect cell cycle regulation and other pathways and could be a fascinating topic for future study. This study provides a valuable contribution to scientists interested in cell fate differentiation mechanisms in plants and the molecular mechanisms behind cell fate differentiation.

## Data Availability Statement

The datasets generated for this study can be found in online repositories. The names of the repository/repositories and accession number(s) can be found in the article/Supplementary Material.

## Author Contributions

HY: conceptualization. CY, SY, and YZ: methodology. SY and YZ: validation. CY, SY, and WY: formal analysis. WJ and XW: data. HY: writing—original draft preparation. WW and XH: writing—review and editing. HY: funding. All authors contributed to the article and approved the submitted version.

## Conflict of Interest

The authors declare that the research was conducted in the absence of any commercial or financial relationships that could be construed as a potential conflict of interest.

## Footnotes

1 http://structuralbiology.cau.edu.cn/PlantGSEA

2 http://revigo.irb.hr/

3 http://string-db.org/

4 http://www.genome.jp/kaas-bin/kaas_main

5 http://planttfdb.gao-lab.org/

6 http://arabidopsis.med.ohio-state.edu/AtTFDB/

7 http://bio-med.biomart.cn/

8 http://arabidopsis.info/

9 www.arashare.cn

## Supplementary Material

Supplementary Figure 1 Diagram of the experimental procedure.

Supplementary Figure 2 The chromatogram of the peptide mixture fractionated by high-pH reversed-phase chromatography.

Supplementary Figure 3 The PPI network of other DAPs highly expressed in VN and GN.

Supplementary Figure 4 The metabolism network of 57 DAPs highly expressed in VN using STRING.

Supplementary Table 1 The summary of MS result.

Supplementary Table 2 Detailed information of 720 DAPs identified by mass spectrometry.

Supplementary Table 3 The functional categories of the 720 DAPs.

Supplementary Table 4 The list of identified transcription factors.
