# Supplementary material for: Proteomic Analysis of Generative and Vegetative Nuclei Reveals Molecular Characteristics of Pollen Cell Differentiation in Lily
Source: Front Plant Sci. 2021 Jun 7;12:641517. doi: 10.3389/fpls.2021.641517 (PMC8215658; doi:10.3389/fpls.2021.641517)
Supplement: Supplementary Figure 1 — Diagram of the experimental procedure. [file Data_Sheet_1.zip › Figure S1.DOCX]

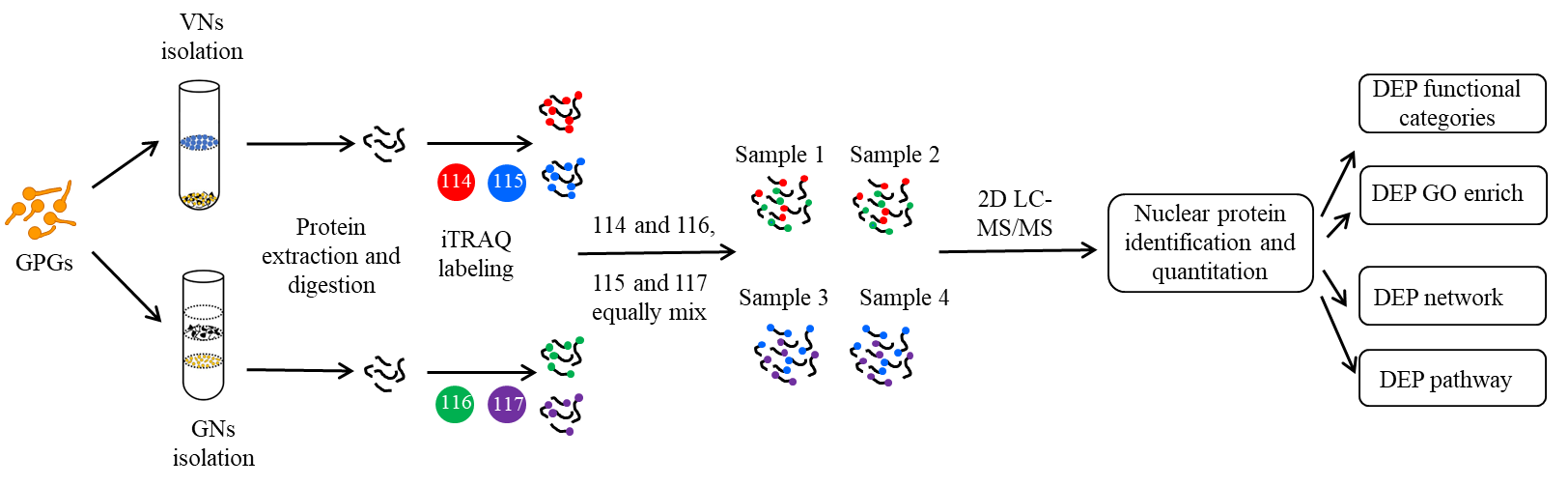


**Figure S1** Sketch of the experiment procedures. VNs and GNs were isolated from germinate pollen grains (GPGs) by percoll density gradient centrifugation. After being digested by trypsin, nuclear protein from VNs were labeled with iTRAQ tags 114 and 115, while that from GNs were labeled with tags 116 and 117. The equally mixed 114 and 116 labeled peptides and 115 and 117 labeled peptides, totally four repeats, were analyzed and identified by two-dimensional liquid chromatography/tandem mass spectrometry (2D LC-MS/MS). At last, the identified DEPs were analyzed using different bioinformatics tools.


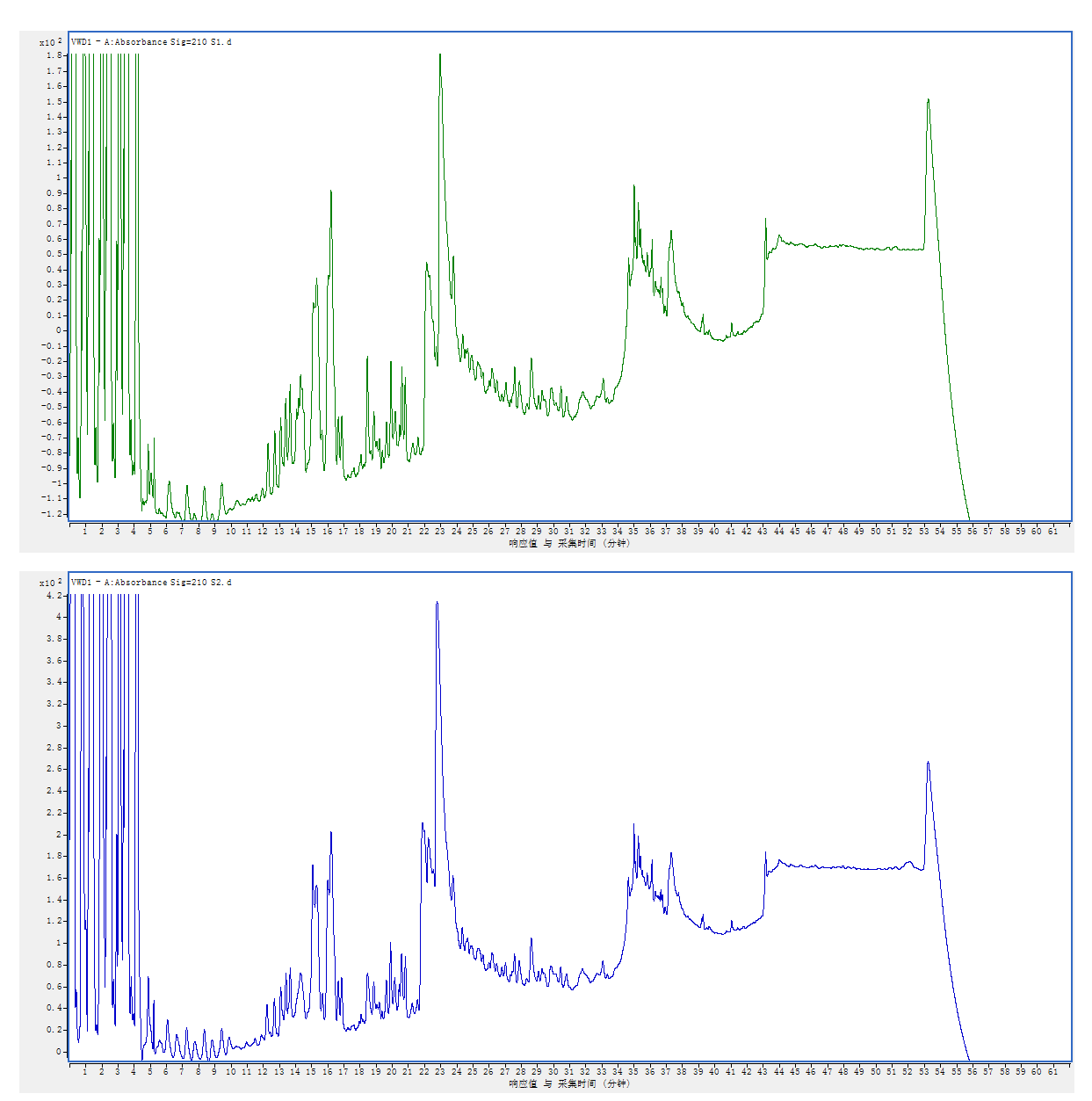


**Figure S2** The chromatogram of the peptide mixture fractionated by high-pH reversed phase chromatography.


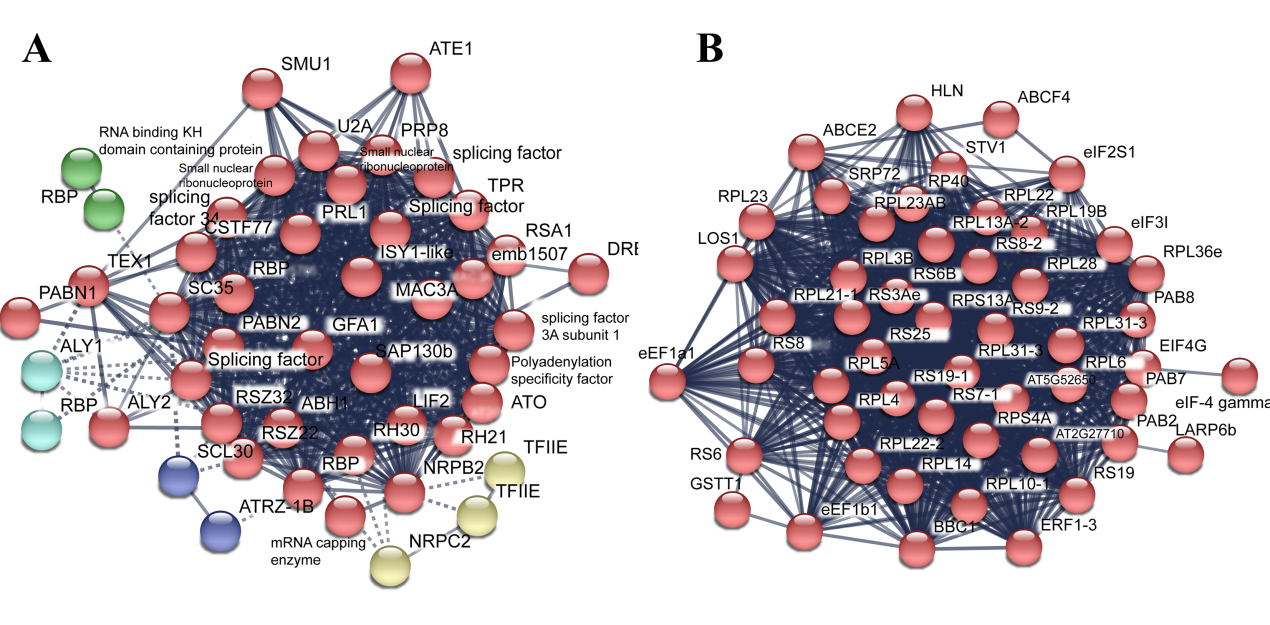
**Figure S3** Protein interaction network analysis of differentially expressed proteins between VN and GN by using Arabidopsis homologue proteins. (A) Protein interaction network analysis of 60 DAPs highly expressed in VN involved in mRNA processing. (B) Protein interaction network analysis of 51 DAPs highly expressed in GN involved in ribosome assembly and translation.


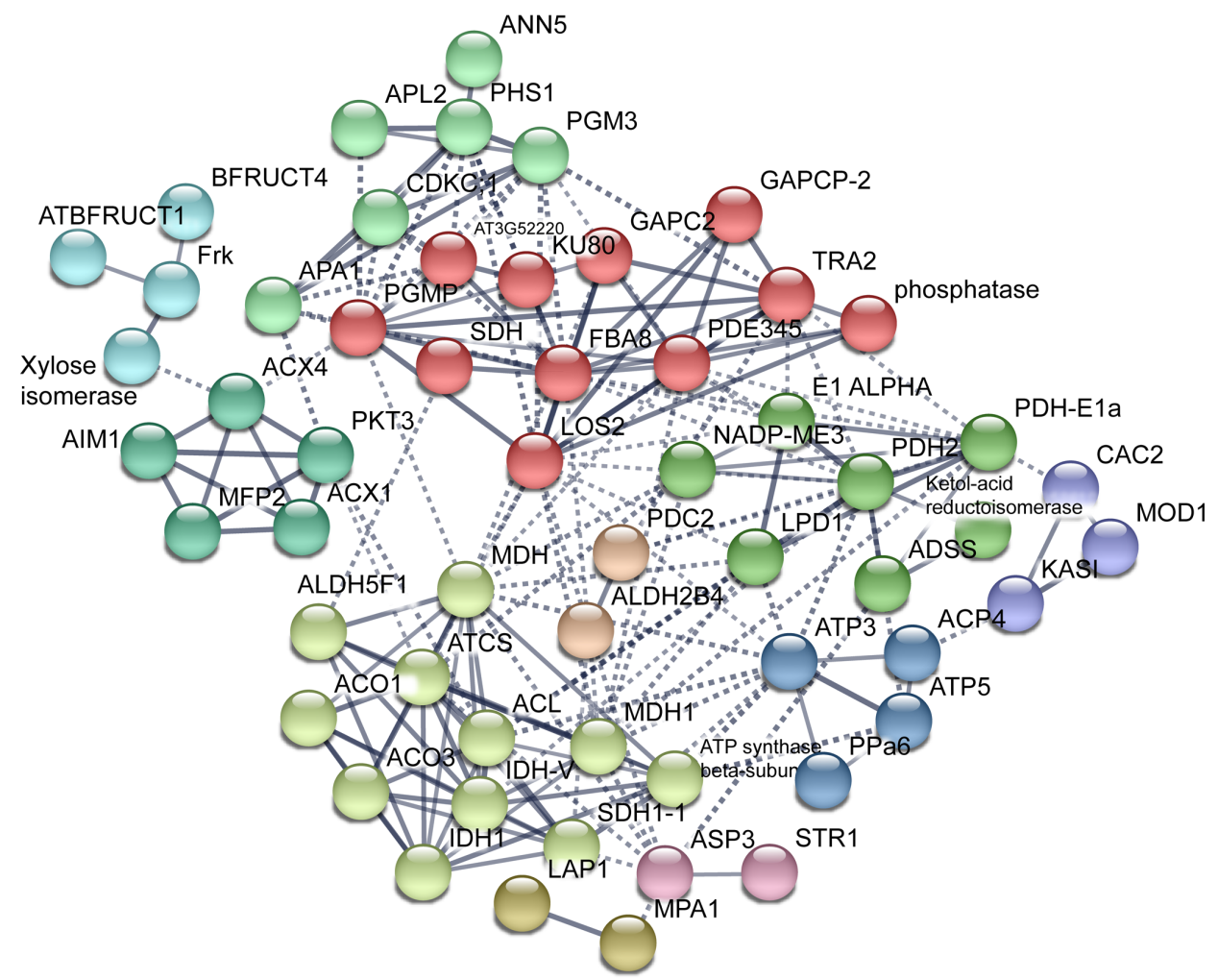


**Figure S4** The metabolism interaction network of 57 DAPs highly expressed in VN using STRING.
