# Supplementary material for: Proteomic Analysis of Generative and Vegetative Nuclei Reveals Molecular Characteristics of Pollen Cell Differentiation in Lily
Source: Front Plant Sci. 2021 Jun 7;12:641517. doi: 10.3389/fpls.2021.641517 (PMC8215658; doi:10.3389/fpls.2021.641517)
Supplement: Supplementary Figure 1 — Diagram of the experimental procedure. [file Data_Sheet_1.zip › Table S1.DOCX]

**Table S1 The summary of MS results**

|  | Total  spectra*^a^* | Identified  peptides (99% conf)*^b^* | Identified  proteins (1% FDR)*^c^* | Identified  proteins (Unused>4)*^d^* | Differential expressed  proteins (≥1.5,≤0.67)*^e^* |
| --- | --- | --- | --- | --- | --- |
| S1 | 186930 | 46297 | 3505 | 1938 | 730 |
| S2 | 186185 | 45739 | 3519 | 1901 | 709 |
| S3 | 191729 | 47143 | 3659 | 1991 | 758 |
| S4 | 190783 | 47443 | 3738 | 2031 | 789 |

*^a^* Total MS spectra acquired from MS instrument. *^b^* Total identified confidence 99% peptides. *^c^*The total identified proteins when used the 1% False discovery rate. *^d^*the total identified proteins when the unused score is greater than 4. *^e^*The change fold ≥ 1.5 (or ≤ 0.67) and p-value <0.05 are as criterion of differential expressed protein.

**Table S3** The functional categories of the 720 DAPs

| RNA-seq ID | Description | *Arabidopsis*  Homologue | | Fold change  (GN/VN) |
| --- | --- | --- | --- | --- |
| Chromatin Structure (35) | | | | |
| Chromatin condensation (18) | | | | |
| Unigene18129 | Structural maintenance of chromosomes 1 | SMC1/TTN8 | AT3G54670 | 7.14 |
| Unigene18130 | Structural maintenance of chromosomes 1 | SMC1/TTN8 | AT3G54670 | 8.95 |
| Unigene20903 | Structural maintenance of chromosomes 2 | SMC2 | AT5G62410 | 6.22 |
| Unigene21503 | Structural maintenance of chromosomes 3 | SMC3 | AT2G27170 | 9.64 |
| Unigene22534 | Structural maintenance of chromosome 3 | SMC3 | AT5G48600 | 9.12 |
| Unigene21504 | Structural maintenance of chromosomes 3 | SMC3 | AT2G27170 | 2.47 |
| Unigene19809 | Structural maintenance of chromosomes 3 | SMC3/TTN7 | AT2G27170 | 7.21 |
| Unigene21505 | Structural maintenance of chromosomes 3 | SMC3/TTN7 | AT2G27170 | 5.38 |
| Unigene22535 | Chromosome associated protein-c | SMC3 | AT5G48600 | 10.67 |
| Unigene25603 | Sister chromatid cohesion 1 protein 3 | SYN3 | AT3G59550 | 7.05 |
| Unigene19796 | Sister chromatid cohesion 1 protein 4 | SYN4 | AT5G16270 | 6.55 |
| CL19.Contig2 | Tudor domains-containing protein | PDS5C | AT4G31880 | 29.06 |
| Unigene20902 | Structural maintenance of chromosomes 2 | SMC2 | AT5G62410 | 7.28 |
| Unigene23361 | Condensin complex subunit 3 | EMB2656 | AT5G37630 | 4.73 |
| Unigene23407 | Structural maintenance of chromosomes 5 | SMC5 | AT5G15920 | 6.45 |
| Unigene16790 | structural maintenance of chromosomes 6B | SMC6B/MIM | AT5G61460 | 3.77 |
| CL4004.Contig2 | structural maintenance of chromosomes domain protein |  | AT3G14750 | 0.47 |
| Unigene17872 | Structural maintenance of chromosomes flexible hinge domain-containing protein | GMI1 | AT5G24280 | 19.06 |
| Chromatin remodeling (17) | | | | |
| CL5160.Contig1 | Chromatin remodeling complex subunit R3 | CHR3 | AT2G28290 | 4.41 |
| Unigene25873 | Chromatin remodeling 12 | CHR12 | AT3G06010 | 2.99 |
| Unigene24596 | Chromatin remodeling 19 | CHR19 | AT2G02090 | 3.56 |
| CL2869.Contig1 | Chromatin remodeling factor17 | CHR17 | AT5G18620 | 2.00 |
| Unigene20975 | Chromatin remodeling protein | EBS | AT4G22140 | 0.16 |
| CL4116.Contig1 | Chromatin remodeling 5 | CHR5 | AT2G13370 | 0.33 |
| CL1630.Contig1 | Topoisomerase 1 beta | TOP1 Beta | AT5G55310 | 0.25 |
| Unigene23313 | Topoisomerase II | TOPII | AT3G23890 | 11.12 |
| Unigene25000 | Topoisomerase 6 subunit B | TOP6 B | AT3G20780 | 13.76 |
| CL4296.Contig2 | Brassinosteroid-insensitive 4 | BIN4 | AT5G24630 | 20.32 |
| Unigene24052 | Topoisomerase 6 | TOP6/ RHL2 | AT5G02820 | 12.75 |
| Unigene14410 | Root hairless 1 | RHL1 | AT1G48380 | 14.01 |
| Unigene2181 | Root hairless 2 | RHL2 | AT5G02820 | 8.24 |
| Unigene22480 | DEK domain-containing chromatin associated protein |  | AT3G48710 | 9.26 |
| Unigene15562 | DEK domain-containing chromatin associated protein |  | AT4G26630 | 12.99 |
| CL1705.Contig1 | SWIB complex BAF60b domain-containing protein |  | AT3G19080 | 4.73 |
| CL5706.Contig1 | TRF-LIKE 2 | TRFL2 | AT1G07540 | 0.20 |
| Nuclear membrane structure (20) | | | | |
| Unigene20026 | Nucleoporin 155 | NUP155 | AT1G14850 | 0.36 |
| Unigene21043 | Embryo Defective 3142 | NUP205 | AT5G51200 | 0.42 |
| CL4053.Contig1 | NUP1 | NUP1 | AT3G10650 | 4.66 |
| CL3210.Contig1 | Nuclear pore complex protein NUP85 | NUP85 | AT4G32910 | 0.37 |
| Unigene21057 | Nucleoprotein TPR | NUA | AT1G79280 | 0.48 |
| Unigene24368 | Nucleoprotein TPR | NUA | AT1G79280 | 0.51 |
| Unigene9598 | Nuclear pore complex protein | EMB3142 | AT5G51200 | 0.58 |
| Unigene24069 | SAD1/UNC-84 domain protein 1 | SUN1 | AT5G04990 | 0.22 |
| Unigene23969 | SAD1/UNC-84 domain protein 1 | SUN1 | AT5G04990 | 0.57 |
| CL820.Contig1 | Little Nuclei 1 | LINC1 | AT1G67230 | 0.38 |
| CL820.Contig2 | Little Nuclei 1 | LINC1 | AT1G67230 | 0.24 |
| Unigene23646 | Little Nuclei 2 | LINC2 | AT1G13220 | 0.31 |
| Unigene23645 | Little Nuclei 3 | LINC3 | AT1G68790 | 0.47 |
| CL5772.Contig1 | Japanese for nucleus 4 | KAKU4 | AT4G31430 | 0.32 |
| Unigene22809 | Ran-binding protein 1-a | RANBP | AT1G07140 | 8.91 |
| Unigene18652 | RAN GTPase activating protein 1 | RANGAP1 | AT3G63130 | 0.24 |
| Unigene18651 | Ran GTPase Activating protein 1 | RANGAP1 | AT3G63130 | 0.21 |
| Unigene19650 | WPP domain-interacting protein 1 | WIT1 | AT5G11390 | 0.14 |
| CL7050.Contig2 | WPP domain-interacting protein 1 | WIT1 | AT5G11390 | 0.09 |
| Unigene19653 | WPP domain-interacting protein 1 | WIT1 | AT5G11390 | 0.21 |
| Transcription process (46) | | | | |
| Transcription factors (29) | | | | |
| Unigene22395 | Cell Division Cycle 5 | CDC5 | AT1G09770 | 0.53 |
| CL5179.Contig2 | HMG box protein with ARID domain |  | AT1G04880 | 0.28 |
| Unigene23227 | Homologue of NAP57 | NAP57 | AT3G57150 | 2.19 |
| Unigene16954 | DNA-binding storekeeper protein-related transcriptional regulator |  | AT3G04930 | 0.31 |
| Unigene21795 | DNA-binding storekeeper protein-related transcriptional regulator |  | AT1G61730 | 6.88 |
| Unigene22672 | DNA-binding storekeeper protein-related transcriptional regulator |  | AT4G00610 | 7.21 |
| Unigene24387 | DNA-binding storekeeper protein-related transcriptional regulator |  | AT5G28040 | 1.95 |
| CL1029.Contig2 | ATROPOS | ATO | AT5G06160 | 0.30 |
| Unigene25241 | PHD type transcription factor with trans-membrane domains | PTM | AT5G35210 | 2.60 |
| Unigene5682 | Nuclear factor Y, subunit B11 | NF-YB11 | AT2G27470 | 4.90 |
| CL4468.Contig1 | Nuclear factor Y, subunit C10 | NF-YC10 | AT1G07980 | 4.27 |
| Unigene621 | RNA-binding family protein | RBP | AT2G19380 | 2.70 |
| Unigene24902 | RWP-RK domain-containing 3 | RKD3 | AT5G66990 | 18.48 |
| CL7121.Contig1 | Alfin-like 3 | AL3 | AT3G42790 | 31.47 |
| Unigene7122 | Alfin-like 3 | AL3 | AT3G42790 | 4.21 |
| CL131.Contig1 | Zinc finger (C2H2 type) family protein | MBS1 | AT3G02790 | 26.23 |
| Unigene16864 | AGAMOUS-like 65 | AGL65 | AT1G18750 | 0.11 |
| Unigene5688 | AGAMOUS-like 104 | AGL104 | AT1G22130 | 0.15 |
| CL2063.Contig3 | RING/U-box superfamily protein |  | AT3G62240 | 3.53 |
| Unigene24793 | Transcription factor-like protein |  | AT4G17020 | 0.09 |
| Unigene16424 | Basic leucine zipper transcription factor-like | BZIP61 | AT3G58120 | 0.24 |
| CL4663.Contig1 | Transcription factor BIM1 |  | AT5G08130 | 6.37 |
| CL1732.Contig1 | Transcription initiation factor TFIIE | TFIIE | AT4G20330 | 0.33 |
| Unigene19852 | Transcription factor TFIIE | TFIIE | AT1G03280 | 0.27 |
| Unigene300 | BTF2-like transcription factor | TFIIH subunit H1 | AT1G55750 | 0.14 |
| Unigene25958 | Asi1-Immunoprecipitated Protein 3 | AIPP3 | AT4G11560 | 0.46 |
| CL900.Contig3 | Asi1-Immunoprecipitated Protein 1 | AIPP1 | AT1G05970 | 5.25 |
| Unigene24317 | Enhanced Downy Mildew 2 | EDM2 | AT5G55390 | 4.90 |
| CL240.Contig3 | Global transcription factor group E8 | GTE8 | AT3G27260 | 3.40 |
| Transcription process (17) | | | | |
| Unigene18751 | Zinc finger C-x8-C-x5-C-x3-H type family protein |  | AT3G18640 | 0.25 |
| CL4379.Contig2 | Zinc finger C-x8-C-x5-C-x3-H type family protein |  | AT3G21810 | 0.48 |
| CL5658.Contig2 | TOPLESS-related 1 | TPR1 | AT1G80490 | 2.48 |
| Unigene18498 | Purin-rich alpha 1 | PURA1 | AT2G32080 | 11.29 |
| CL6080.Contig2 | FAR1-related sequence 11 | FRS11 | AT1G10240 | 0.47 |
| CL4277.Contig2 | DNA-directed RNA polymerase II subunit RPB2 | NRPB2 | AT4G21710 | 0.48 |
| Unigene17609 | Nuclear RNA polymerase C2 | NRPC2 | AT5G45140 | 0.57 |
| CL6986.Contig1 | De-capping nuclease DXO homolog |  | AT4G17620 | 0.28 |
| Unigene11311 | Root Growth Defective 3 | RGD3/BTAF1 | AT3G54280 | 0.12 |
| Unigene26687 | Root Growth Defective 3 | RGD3/BTAF1 | AT3G54280 | 0.17 |
| Unigene5528 | Root Growth Defective 3 | RGD3/BTAF1 | AT3G54280 | 0.23 |
| Unigene12350 | Root Growth Defective 3 | RGD3/BTAF1 | AT3G54280 | 0.30 |
| Unigene21608 | 14-3-3-like protein | 14-3-3ν | AT3G02520 | 4.11 |
| CL6161.Contig1 | General Regulatory Factor 12 | GRF12 | AT1G26480 | 4.97 |
| CL5526.Contig3 | Transcript elongation factor IIS | TFIIS | AT2G38560 | 2.05 |
| CL3539.Contig3 | RING/FYVE/PHD zinc finger containing protein |  | AT3G20280 | 5.91 |
| CL5195.Contig1 | Multiprotein bridging factor 1A | MBF1A | AT2G42680 | 20.35 |
| Translation process (62) | | | | |
| Ribosome assembly (44) | | | | |
| CL1546.Contig3 | Ribosomal protein L3 B | RPL3B | AT1G61580 | 2.55 |
| CL4104.Contig1 | Ribosomal protein large subunit 16a | RPL16A | AT2G42740 | 2.91 |
| CL5973.Contig2 | Ribosomal protein L5 | RPL5 | AT3G25520 | 3.96 |
| CL50.Contig2 | Suppressor of Acaulis 56 | RPL4 | AT3G09630 | 4.17 |
| Unigene15137 | Ribosomal protein L10 | RPL10-1 | AT2G40010 | 3.98 |
| Unigene20856 | Ribosomal protein L36e |  | AT3G53740 | 3.16 |
| CL1197.Contig1 | Ribosomal protein L13 | RPL13/BBC1 | AT3G49010 | 3.75 |
| CL6771.Contig2 | Ribosomal protein L13 | RPL13A-2 | AT3G24830 | 3.04 |
| CL4997.Contig3 | Ribosomal protein L23A2 | RPL23A2 | AT3G55280 | 3.63 |
| CL2709.Contig3 | Ribosomal protein L6 |  | AT1G74050 | 3.25 |
| CL1820.Contig2 | Ribosomal protein L14 |  | AT4G27090 | 4.57 |
| CL5176.Contig1 | Translation protein SH3-like | RPL21-1 | AT1G09590 | 2.63 |
| CL2624.Contig1 | Ribosomal protein like 19B | RPL19B | AT3G16780 | 2.38 |
| CL5377.Contig2 | Ribosomal L22e | RPL22-2 | AT3G05560 | 5.71 |
| CL6496.Contig1 | Short Valve1 | STV1/ RPL24 | AT3G53020 | 6.14 |
| CL1903.Contig1 | Ribosomal L28e |  | AT4G29410 | 16.29 |
| Unigene7030 | 60S acidic ribosomal protein family |  | AT2G27710 | 3.87 |
| CL1197.Contig2 | 40S Ribosomal Protein | ATBBC1 | AT3G49010 | 7.45 |
| CL4729.Contig2 | Ribosomal protein L31e | RPL31-3 | AT5G56710 | 14.46 |
| CL3589.Contig2 | Ribosomal protein L22p |  | AT1G67430 | 5.35 |
| CL266.Contig2 | Ribosomal protein L14p/L23e | RPL23 | AT1G04480 | 7.77 |
| Unigene12167 | Ribosomal protein L7Ae/L30e/S12e/Gadd45 | RPL | AT5G08180 | 3.33 |
| CL3627.Contig1 | SAR DNA-binding protein |  | AT3G05060 | 4.05 |
| CL2122.Contig2 | S10 domain-containing protein |  | AT5G52650 | 8.17 |
| CL4641.Contig1 | Ribosomal protein S4 | RS9-2 | AT5G39850 | 2.73 |
| CL1110.Contig4 | Ribosomal protein S4 | RPS4A | AT5G58420 | 7.94 |
| Unigene15745 | Ribosomal protein S5 |  | AT1G59359 | 12.71 |
| CL2340.Contig1 | Ribosomal protein S3Ae |  | AT4G34670 | 5.01 |
| CL5585.Contig4 | Ribosomal protein S6b | RS6B | AT5G10360 | 6.23 |
| Unigene22394 | Ribosomal protein S6 |  | AT2G18110 | 6.43 |
| Unigene19037 | Ribosomal protein S7-1 | RS7-1 | AT1G48830 | 7.09 |
| CL767.Contig7 | Ribosomal protein S8e | RS8-2 | AT5G59240 | 5.25 |
| CL3197.Contig3 | Ribosomal protein S8 |  | AT5G59850 | 9.82 |
| CL5420.Contig1 | Ribosomal protein S13a | RPS13A | AT4G00100 | 5.81 |
| Unigene10851 | Ribosomal protein S19e | RS19-1 | AT3G02080 | 7.24 |
| CL4749.Contig2 | Ribosomal protein S19 |  | AT5G09500 | 9.73 |
| CL1362.Contig1 | Ribosomal protein S25 |  | AT4G34555 | 5.97 |
| CL4846.Contig1 | 40s Ribosomal protein SA | RP40 | AT1G72370 | 9.43 |
| Unigene21794 | Nucleolin | NUC-L2 | AT3G18610 | 1.89 |
| Unigene23245 | Homolog of nucleolar protein nop56 | NOP56 | AT1G56110 | 3.63 |
| Unigene80 | DOMINO1 |  | AT5G62440 | 2.10 |
| Unigene7779 | Alba DNA/RNA-binding protein |  | AT2G34160 | 16.75 |
| Unigene24231 | Alba DNA/RNA-binding protein |  | AT1G76010 | 16.73 |
| CL7065.Contig1 | ATP-binding cassette E2 | ABCE2 | AT4G19210 | 3.10 |
| Translation (18) | | | | |
| Unigene17343 | Translation initiation factor 2 subunit 1 | eIF2S1 | AT2G40290 | 5.78 |
| Unigene20361 | Translation initiation factor eIF-4F | eIF4G | AT3G60240 | 5.70 |
| CL6035.Contig1 | Translation initiation factor eIF-2 beta subunit | eIF-2BS | AT5G20920 | 9.56 |
| Unigene682 | Translation initiation factor SUI1-like protein | SUI1-like | AT1G71350 | 6.13 |
| Unigene20296 | Translation initiation factor SUI1-like protein |  | AT5G11900 | 4.87 |
| Unigene25768 | Translation initiation factor | IF-3 | AT1G34360 | 0.48 |
| Unigene15073 | Translation initiation factor 3 subunit I | eIF3i | AT2G46290 | 4.92 |
| Unigene23029 | MA3 domain-containing protein | eIF-4 gamma | AT1G22730 | 9.96 |
| CL885.Contig3 | MA3 domain-containing translation regulatory factor 3 | MRF3 | AT4G24800 | 3.70 |
| Unigene22393 | Translation elongation factor EF1B | eEF1b1 | AT5G12110 | 7.12 |
| CL522.Contig1 | Elongation factor 2 | LOS1 | AT1G56070 | 4.09 |
| Unigene26301 | Transcription elongation factor | TFIIS | AT4G24200 | 0.23 |
| CL472.Contig4 | GTP binding Elongation factor Tu family protein | eEF1a1 | AT1G07920 | 14.54 |
| CL3577.Contig2 | Eukaryotic release factor 1-3 | ERF1-3 | AT3G26618 | 2.78 |
| Unigene20627 | Nucleic acid-binding, OB-fold-like |  | AT2G04520 | 9.13 |
| Unigene19357 | Nucleic acid-binding, OB-fold-like |  | AT1G23750 | 9.68 |
| CL756.Contig2 | Nucleic acid-binding, OB-fold-like |  | AT5G03850 | 7.94 |
| Unigene23148 | Developmentally-regulated G-protein 3 | DRG3 | AT4G39520 | 3.66 |
| RNA processing (108) | | | | |
| mRNA processing (27) | | | | |
| Unigene22310 | poly(A) binding protein 2 | PAB2 | AT4G34110 | 6.34 |
| CL1923.Contig2 | poly(A) binding protein 4 | PAB4 | AT2G23350 | 9.70 |
| Unigene18932 | poly(A) binding protein 7 | PAB7 | AT2G36660 | 7.24 |
| CL3001.Contig1 | Poly(A) binding protein 8 | PAB8 | AT1G49760 | 6.22 |
| Unigene24564 | RNA-binding protein 45B | RBP45B | AT1G11650 | 10.47 |
| Unigene20585 | RNA-binding protein 45B | RBP45B | AT1G11650 | 1.75 |
| CL5243.Contig2 | Poly(A) binding protein 2 | PAB2 | AT4G34110 | 6.85 |
| Unigene23938 | Evolutionarily conserved c-terminal region 2 | ECT2 | AT3G13460 | 2.99 |
| CL1163.Contig3 | Evolutionarily conserved c-terminal region 2 | ECT2 | AT3G13460 | 6.19 |
| CL6042.Contig1 | Polyadenylate-binding protein 2 | PABN2 | AT5G65260 | 0.17 |
| Unigene22192 | Polyadenylate-binding protein | RBP47B | AT5G19350 | 0.25 |
| Unigene6526 | Polyadenylate-binding protein 1 | PABN1 | AT5G51120 | 0.20 |
| Unigene19334 | ENTH/VHS family protein |  | AT3G26990 | 0.37 |
| CL1553.Contig1 | pre-mRNA-processing protein 40A | PRP40A | AT1G44910 | 0.38 |
| CL1553.Contig3 | pre-mRNA-processing protein 40A | PRP40A | AT1G44910 | 0.37 |
| CL5822.Contig1 | pre-mRNA-processing 8 | PRP8 | AT1G80070 | 0.50 |
| CL2405.Contig2 | mRNA capping enzyme |  | AT3G09100 | 0.39 |
| CL4037.Contig2 | Nuclear cap-binding protein subunit 1 | ABH1 | AT2G13540 | 0.25 |
| Unigene20210 | THO complex subunit 4A | ALY1 | AT5G59950 | 0.42 |
| Unigene6916 | THO complex subunit 3 | TEX1 | AT5G56130 | 0.51 |
| Unigene29 | THO complex subunit 4B | ALY2 | AT5G02530 | 0.47 |
| CL2346.Contig2 | HAKAI |  | AT5G01160 | 3.94 |
| CL2404.Contig1 | Embryo defective 1691 | EMB1691 | AT4G09980 | 6.97 |
| Unigene23509 | Protein Virilizer homolog | VIR | AT3G05680 | 4.90 |
| Unigene22679 | FKBP12 interacting protein 37 | FIP37 | AT3G54170 | 6.04 |
| CL2279.Contig2 | Apoptosis inhibitory protein 5 | API5 | AT2G34040 | 0.26 |
| CL5103.Contig2 | ATPase E1 | ATE1 | AT1G13690 | 0.30 |
| mRNA Splicing (52) | | | | |
| CL6734.Contig2 | Splicing factor 3B subunit 3 | SAP130B | AT3G55220 | 0.47 |
| CL1310.Contig2 | Splicing factor |  | AT5G64270 | 0.34 |
| Unigene20798 | Polyadenylation specificity factor |  | AT4G25550 | 0.33 |
| Unigene18882 | Splicing factor | SC35 | AT5G64200 | 0.19 |
| CL1196.Contig1 | Splicing factor PWI domain containing protein |  | AT2G29210 | 0.14 |
| CL3827.Contig2 | Putative splicing factor |  | AT2G43770 | 0.26 |
| Unigene18979 | Probable splicing factor 3A subunit 1 |  | AT1G14650 | 0.40 |
| Unigene21494 | Cleavage stimulation factor 77 | CSTF77 | AT1G17760 | 0.31 |
| Unigene26249 | pre-mRNA-splicing factor ISY1-like |  | AT3G18790 | 0.47 |
| CL5901.Contig2 | SC35-like splicing factor 30 | SCL30 | AT3G55460 | 0.65 |
| CL2150.Contig2 | serine/arginine rich splicing factor | RSZ22 | AT4G31580 | 0.22 |
| CL6775.Contig1 | serine/arginine rich splicing factor 34 |  | AT3G49430 | 0.25 |
| Unigene23286 | Serine/arginine rich splicing factor 34 | SRP34 | AT1G02840 | 1.72 |
| CL6567.Contig1 | Serine/arginine-rich splicing factor | RS2Z33 | AT2G37340 | 3.56 |
| Unigene22484 | Serine/arginine-rich splicing factor | RSZ32 | AT3G53500 | 0.41 |
| CL1419.Contig4 | Tetratricopeptide repeat-like protein | PRP39 | AT1G04080 | 0.24 |
| Unigene24354 | Tetratricopeptide repeat-like protein | TPR | AT5G28740 | 0.46 |
| CL5429.Contig1 | LUC7 related protein | LUC7B | AT5G17440 | 0.30 |
| CL5429.Contig2 | Unfertilized embryo sac 6 | UNE6 | AT3G03340 | 0.32 |
| Unigene8728 | Suppressors Of Mec-8 and Unc-52 | SMU1 | AT1G73720 | 0.42 |
| Unigene23010 | Spliceosome associated protein 130 | SAP130B | AT3G55220 | 0.33 |
| Unigene21980 | DEAD-box RNA helicase 21 | RH21 | AT2G33730 | 0.49 |
| CL1774.Contig1 | DEAD box RNA helicase 1 | DRH1 | AT3G01540 | 0.49 |
| CL1859.Contig5 | DEAD-box RNA helicase 30 | RH30 | AT5G63120 | 0.19 |
| Unigene183 | DEAD-box RNA helicase 38 | RH38 | AT3G53110 | 4.87 |
| Unigene25046 | DEAD box RNA helicase | RH40 | AT3G06480 | 0.31 |
| Unigene20420 | DEAD box RNA helicase | HVT1 | AT2G30800 | 0.28 |
| CL399.Contig1 | RNA Helicase 37 | RH37 | AT2G42520 | 3.35 |
| Unigene24994 | DEAD-box RNA helicase 56 | UAP56B | AT5G11200 | 4.92 |
| CL420.Contig1 | DEAD box RNA helicase DExH12 | EMB1507 | AT1G20960 | 0.35 |
| CL420.Contig6 | DExH-box RNA helicase | BRR2A | AT1G20960 | 0.33 |
| CL2567.Contig1 | Pre-mRNA-splicing factor RNA helicase DEAH1 | ESP3 | AT1G32490 | 3.28 |
| Unigene157 | CwfJ-like family protein |  | AT1G56290 | 5.35 |
| CL608.Contig2 | Cyclophilin-like peptidyl-prolyl cis-trans isomerase family protein |  | AT3G63400 | 0.36 |
| Unigene22510 | Nucleotide binding protein |  | AT1G21320 | 0.13 |
| CL6644.Contig1 | Nucleotide binding protein | NSRB | AT1G21320 | 0.56 |
| Unigene219 | Small nuclear ribonucleoprotein |  | AT5G44500 | 0.30 |
| CL6510.Contig3 | Small nuclear ribonucleoprotein |  | AT3G62840 | 0.30 |
| Unigene19129 | Small nuclear ribonucleoprotein |  | AT1G20580 | 0.42 |
| Unigene17264 | U2 small nuclear ribonucleoprotein A | U2A | AT1G09760 | 0.30 |
| Unigene24033 | UBP1-associated protein 2B | UBA2B | AT2G41060 | 0.24 |
| Unigene20561 | UBP1-associated protein 2A | UBA2A | AT3G56860 | 0.16 |
| Unigene11142 | UBP1-associated protein 2C | UBA2C | AT3G15010 | 0.27 |
| Unigene21944 | RNA-binding family protein | RBP | AT4G35785 | 0.22 |
| Unigene8624 | Glycine-rich RNA-binding protein 7 | GRP7 | AT2G21660 | 19.59 |
| Unigene21986 | RNA-binding glycine rich protein d4 | RBGD4 | AT4G14300 | 3.16 |
| Unigene8454 | RNA-binding glycine rich protein d4 | RBGD4 | AT4G14300 | 5.40 |
| Unigene20272 | RNA-binding glycine rich protein d3 | RBGD3 | AT3G13224 | 0.23 |
| Unigene19990 | RNA-binding family protein | RBP | AT1G76940 | 0.21 |
| Unigene14417 | RNA-binding family protein | RBP | AT1G76940 | 0.25 |
| Unigene17682 | CLP-SIMILAR PROTEIN 3 | CLPS3 | AT3G04680 | 7.73 |
| Unigene23594 | Reduced Red-Light Responses In Cry1cry2 Background 1 | RRC1 | AT5G25060 | 2.27 |
| mRNA binding (29) | | | | |
| Unigene25051 | RNA-binding family protein | RBP | AT2G44710 | 0.24 |
| Unigene25050 | RNA-binding family protein | RBP | AT2G44710 | 0.18 |
| Unigene21783 | RNA-binding family protein | RBP | AT2G44710 | 0.50 |
| Unigene23003 | RNA-binding family protein | RBP | AT2G44710 | 0.19 |
| CL1593.Contig1 | RNA-binding family protein | RBP | AT2G44710 | 0.27 |
| Unigene22407 | RNA-binding family protein | RBP | AT2G44710 | 0.33 |
| CL2129.Contig1 | RNA-binding family protein | RBP | AT2G44710 | 0.24 |
| Unigene21082 | RNA-binding family protein | RBP | AT3G20890 | 0.26 |
| CL5289.Contig4 | RNA-binding family protein | RBP | AT3G07810 | 0.20 |
| Unigene14931 | RNA-binding family protein | RBP | AT3G13700 | 0.23 |
| Unigene23237 | RNA-binding family protein | RBP | AT4G36960 | 0.19 |
| Unigene980 | RNA-binding family protein | RBP | AT5G19960 | 0.27 |
| CL6766.Contig1 | Ran BP2 zinc finger-like | zf-RanBP | AT1G67325 | 0.19 |
| Unigene14912 | Ran BP2 zinc finger-like | zf-RanBP | AT1G67325 | 0.26 |
| Unigene24946 | RNA recognition motif-containing |  | AT3G23900 | 0.17 |
| Unigene24014 | RNA binding KH domain containing |  | AT5G15270 | 0.33 |
| CL4724.Contig1 | RNA binding KH domain containing |  | AT5G15270 | 0.16 |
| Unigene11949 | RNA binding family protein | RBP | AT3G52660 | 2.54 |
| CL2478.Contig2 | RNA binding KH domain containing |  | AT5G46190 | 0.24 |
| Unigene24013 | RNA binding KH domain containing |  | AT5G46190 | 0.38 |
| CL794.Contig1 | RNA binding KH domain containing |  | AT1G51580 | 0.25 |
| Unigene19353 | RNA binding KH domain containing |  | AT4G10070 | 0.13 |
| Unigene17578 | RNA binding KH domain containing | PEP | AT4G26000 | 0.32 |
| Unigene25170 | Dentin sialophosphoprotein |  | AT3G13990 | 6.19 |
| CL3896.Contig2 | mRNA binding family protein | HLN | AT4G17520 | 9.91 |
| Unigene22633 | mRNA binding family protein | HLN | AT4G17520 | 2.42 |
| Unigene8712 | RNA-binding protein-defense related 1 | BRN1 | AT4G03110 | 2.68 |
| Unigene21109 | RNA-binding protein | LARP6B | AT2G43970 | 7.70 |
| Unigene20290 | RNA binding protein |  | AT2G02570 | 3.94 |
| Cytoskeleton (16) | | | | |
| Unigene23180 | Kinesin 5 | ATK5 | AT4G05190 | 11.72 |
| CL4233.Contig2 | Kinesin-13a |  | AT3G16630 | 4.45 |
| Unigene24050 | Kinesin motor family protein |  | AT3G20150 | 7.11 |
| CL2135.Contig2 | ATP binding microtubule motor |  | AT5G02370 | 12.47 |
| CL896.Contig7 | Tubulin beta-1 chain | TUB1 | AT1G75780 | 13.30 |
| Unigene25002 | P-loop containing nucleoside triphosphate hydrolases | KIF11 | AT2G36200 | 11.96 |
| Unigene7318 | P-loop containing nucleoside triphosphate hydrolases |  | AT5G67630 | 1.80 |
| Unigene25140 | P-loop containing nucleoside triphosphate hydrolases | KIF | AT5G60930 | 6.90 |
| CL1367.Contig2 | Microtubule-associated protein 65-1 | MAP65-1 | AT5G55230 | 23.33 |
| CL4190.Contig1 | Microtubule-associated protein 65-3 | MAP65-3 | AT5G51600 | 6.61 |
| CL238.Contig4 | Microtubule end binding protein 1C | EB1C | AT5G67270 | 17.22 |
| CL238.Contig1 | EB1-homolog1 | EB1C | AT5G67270 | 26.06 |
| Unigene20444 | Tortifolia 1 | TOR1/ SPR2 | AT4G27060 | 6.37 |
| CL7421.Contig3 | Actin 7 | ACT7 | AT5G09810 | 12.35 |
| CL4465.Contig1 | V-ATPase b subunit 2 | VAB2 | AT4G38510 | 0.51 |
| CL1263.Contig2 | Dynamin Related Protein 4c | DRP4C | AT1G60500 | 4.57 |
|  | **Cell fate determinants (10)** | | | |
| Unigene19029 | ENHANCER OF AG-4 1 | HUA1 | AT3G12680 | 8.93 |
| Unigene15085 | U5 small nuclear ribonucleoprotein component | GFA1 | AT1G06220 | 0.20 |
| Unigene6650 | U5 small nuclear ribonucleoprotein component | GFA1 | AT1G06220 | 0.49 |
| Unigene21815 | DUO1-activated ATPase 1 | DAA1 | AT1G64110 | 7.94 |
| CL4279.Contig1 | D Nuclduo1-Activateeic Acid Binding Protein 1 | DAN1 | AT3G04620 | 14.14 |
| Unigene17920 | Histone-lysine N-methyltransferase | SET1 | AT2G23380 | 0.10 |
| Unigene23904 | Microtubule Organization 1/ GEM1 | MOR1 | AT2G35630 | 4.43 |
| Unigene23096 | PDS5 cohesion cofactor | PDS5C | AT4G31880 | 1.63 |
| CL4067.Contig2 | PDS5 cohesion cofactor | PDS5C | AT4G31880 | 8.32 |
| Unigene25058 | PDS5 cohesion cofactor | PDS5C | AT1G77600 | 4.25 |
|  | **Flowering (24)** | | | |
| CL4695.Contig3 | FRIGIDA-like protein |  | AT5G48385 | 0.13 |
| Unigene16768 | FT interacting protein 3 | FTIP3 | AT3G57880 | 6.03 |
| Unigene23038 | Flowering locus KH domain | FLK | AT3G04610 | 0.18 |
| Unigene22943 | Vernalization independence 4 | VIP4 | AT5G61150 | 1.71 |
| Unigene8589 | Vernalization independence 3 | VIP3 | AT4G29830 | 0.28 |
| Unigene22597 | RNA-binding family protein | HLP1 | AT5G40490 | 0.18 |
| CL7176.Contig1 | FRIGIDA-like protein |  | AT5G48385 | 0.13 |
| CL7451.Contig2 | FRIGIDA-like protein |  | AT3G22440 | 0.17 |
| CL7052.Contig2 | FRIGIDA-like protein |  | AT3G22440 | 0.11 |
| Unigene21661 | FRIGIDA-like protein |  | AT3G22440 | 0.18 |
| CL6617.Contig1 | FRIGIDA-ESSENTIAL 1 | FES1 | AT2G33835 | 0.47 |
| Unigene23715 | FLX-like |  | AT3G14750 | 0.40 |
| CL759.Contig1 | FLX-like |  | AT1G67170 | 0.44 |
| Unigene16272 | FLX-like |  | AT1G55170 | 0.40 |
| Unigene22556 | FLX-like |  | AT1G67170 | 0.55 |
| Unigene22555 | FLX-like |  | AT1G67170 | 0.46 |
| Unigene8627 | KH domain-containing protein / zinc finger (CCCH type) family protein | KHZ2 | AT5G06770 | 0.34 |
| Unigene8626 | KH domain-containing protein / zinc finger (CCCH type) family protein | KHZ2 | AT5G06770 | 0.22 |
| CL708.Contig1 | KH domain-containing protein / zinc finger (CCCH type) family protein |  | AT3G12130 | 7.59 |
| Unigene23186 | Early Flowering 8 | ELF8 | AT2G06210 | 0.34 |
| Unigene23025 | Early Flowering 8 | ELF8 | AT2G06210 | 0.21 |
| Unigene11964 | CCT motif family protein |  | AT2G33350 | 0.36 |
| Unigene21725 | Plant homologous to parafibromin | PHP | AT3G22590 | 0.27 |
| Unigene10835 | Flowering time control protein | FPA | AT2G43410 | 0.36 |
| DNA Methylation (25) | | | | |
| DNA Methylation (19) | | | | |
| CL211.Contig9 | S-adenosylmethionine synthase 2 | SAM-2 | AT4G01850 | 3.65 |
| CL601.Contig1 | S-adenosyl-L-methionine-dependent methyltransferases superfamily protein | PMT18 | AT1G33170 | 0.49 |
| CL3185.Contig5 | Anti-silencing 1 | ASI1 | AT5G11470 | 5.70 |
| Unigene103 | DNA-directed RNA polymerases IV and V subunit 4 | RDM2 | AT4G15950 | 27.16 |
| Unigene23929 | DNA-directed RNA polymerases IV and V subunit 2 | NRPD2A | AT3G23780 | 6.55 |
| CL2877.Contig1 | DNA-directed RNA polymerases II, IV and V subunit 11 | NRPD11 | AT3G52090 | 3.34 |
| CL1130.Contig2 | DNA-directed RNA polymerases II, IV and V subunit 3 | NRPB3 | AT2G15430 | 3.66 |
| Unigene21269 | DNA-directed RNA polymerases IV and V subunit 4 | RDM2 | AT4G15950 | 25.12 |
| CL3704.Contig1 | DNA-directed RNA polymerase V subunit 1 | NRPD1B | AT2G40030 | 12.78 |
| Unigene23011 | DNA-directed RNA polymerase V subunit 5A | NRPE5A | AT3G57080 | 7.73 |
| Unigene21562 | Bromo-domain and ATPase domain-containing protein 1 | BRAT1 | AT1G05910 | 4.04 |
| Unigene22150 | Bromodomain-containing protein |  | AT1G58025 | 4.19 |
| CL981.Contig1 | S-adenosyl-l-homocystein hydrolase 1 | SAH1 | AT4G13940 | 0.13 |
| Unigene8483 | Argonaute 4 | AGO4 | AT2G27040 | 15.35 |
| Unigene19540 | Argonaute 4 | AGO4 | AT2G27040 | 4.14 |
| Unigene24627 | Methyl-CPG-binding domain 10 | MBD10 | AT1G15340 | 0.04 |
| Unigene1346 | Methyl-CPG-binding domain 10 | MBD10 | AT1G15340 | 0.12 |
| Unigene21276 | Methyl-CPG-binding domain 11 | MBD11 | AT3G15790 | 0.06 |
| CL6341.Contig1 | Protein MICRORCHIDIA 7 | MORC7 | AT4G24970 | 6.17 |
| miRNA and siRNA biogenesis (6) | | | | |
| Unigene23961 | STABILIZED 1 | STA1 | AT4G03430 | 6.50 |
| CL3012.Contig2 | Suppressor of gene silencing 3 | SGS3 | AT5G23570 | 5.65 |
| Unigene17180 | Double stranded RNA binding protein 4 | DRB4 | AT3G62800 | 0.23 |
| Unigene17178 | dsRNA-binding protein 1 | DRB1 | AT1G09700 | 0.13 |
| Unigene322 | 5'-3' exoribonuclease 3 | XRN3 | AT1G75660 | 0.20 |
| CL837.Contig2 | RNA recognition motif XS domain protein | MCB17.18 | AT3G22430 | 0.47 |
| Histone (44) | | | | |
| Histone variants (12) | | | | |
| CL2845.Contig1 | Histone H1.3 | H1 | AT2G30620 | 42.00 |
| Unigene23759 | Histone H1.1 | H1.1 | CUT18433.1 | 3.40 |
| Unigene18973 | gH2A.1 | HTA2 | AT4G27230 | 30.61 |
| CL549.Contig2 | Histone H2A 2 | HTA2 | AT4G27230 | 35.97 |
| Unigene1309 | Histone H2A 6 | HTA6 | AT5G59870 | 0.19 |
| Unigene23483 | H2A.W.2 | HTA7 | AT5G27670 | 11.33 |
| Unigene25544 | H2A.X.2 | Gamma-H2AX | AT1G54690 | 5.92 |
| Unigene17749 | mgH2B.in | Histone | AT5G02570 | 20.61 |
| CL2535.Contig1 | H2B.4 | HTB1 | AT1G07790 | 4.00 |
| CL542.Contig2 | Histone 3.3 |  | AT4G40030 | 9.73 |
| CL2634.Contig2 | mgH4 | Histone | AT1G07660 | 31.57 |
| Unigene26277 | Centromere protein C | cenH3 | AT1G15660 | 6.55 |
| Histone modification (17) | | | | |
| CL3008.Contig2 | Histone de-acetylation complex 1 | HDC1 | AT5G08450 | 1.87 |
| Unigene21223 | Histone deacetylase 2B | HD2B | AT5G22650 | 2.42 |
| Unigene15693 | Histone deacetylase 2A |  | AT3G44750 | 2.83 |
| Unigene21224 | Histone deacetylase 3 | HDT3 | AT5G03740 | 4.33 |
| Unigene22948 | NAD-dependent protein deacetylase | SRT1 | AT5G55760 | 3.40 |
| Unigene23477 | Transcription factor jumonji (jmjC) domain-containing protein |  | AT1G62310 | 6.37 |
| CL135.Contig2 | Methyltransferase 2 | MET2 | AT5G49160 | 5.30 |
| CL2373.Contig2 | SU(VAR)3-9 homolog 1 | SUVH1 | AT5G04940 | 0.37 |
| Unigene21177 | SU(VAR)3-9 homolog 5 | SUVH5 | AT2G35160 | 5.11 |
| CL1085.Contig3 | SU(VAR)3-9 homolog 5 | SUVH5 | AT2G35160 | 0.42 |
| Unigene345 | Tudor/PWWP/MBT protein |  | AT3G09670 | 0.20 |
| CL6352.Contig1 | Tudor/PWWP/MBT protein |  | AT5G27650 | 4.13 |
| CL5467.Contig1 | Tudor/PWWP/MBT protein | MPO12.6 | AT5G40340 | 2.51 |
| CL1125.Contig1 | Like Heterochromatin Protein 1 | LHP1 | AT5G17690 | 0.22 |
| CL2129.Contig2 | LHP1-interacting factor 2 | LIF2 | AT4G00830 | 0.29 |
| CL3362.Contig1 | LHP1-interacting factor 2 | LIF2 | AT4G00830 | 0.36 |
| Unigene24936 | ABAP1-interacting protein 1 | AIP1 | AT3G62300 | 6.37 |
| Histone chaperone (14) | | | | |
| Unigene1409 | Ubinuclein 1 | UBN1 | AT1G21610 | 2.78 |
| CL6632.Contig2 | Nucleosome assembly protein1;1 | NAP1;1 | AT4G26110 | 13.71 |
| Unigene20745 | NAP1-related protein 2 | NRP2 | AT1G18800 | 0.39 |
| CL5457.Contig2 | Nucleosome Assembly Protein 1;2 | NAP1;2 | AT2G19480 | 6.73 |
| Unigene23868 | Nucleosome Assembly Protein 1;3 | NAP1;3 | AT5G56950 | 8.95 |
| Unigene25726 | HIRA | HIRA | AT3G44530 | 2.94 |
| Unigene22477 | FK506 binding protein 53 | FKBP53 | AT4G25340 | 6.43 |
| Unigene19678 | High mobility group B3 | HMGB3 | AT1G20696 | 13.13 |
| Unigene14399 | High mobility group B1 | HMGB1 | AT3G51880 | 26.59 |
| Unigene26501 | High mobility group B1 | HMGB1 | AT3G51880 | 0.29 |
| CL774.Contig1 | High mobility group | SSRP1 | AT3G28730 | 2.79 |
| CL562.Contig2 | Histone-binding protein RBBP4 | FVE | AT2G19520 | 13.24 |
| CL6845.Contig1 | Tetratricopeptide repeat-like protein | TPR protein | AT4G37210 | 2.48 |
| Unigene24314 | Bucentaur protein | YETI-like | AT5G30490 | 4.81 |
| Cell cycle (15) | | | | |
| Unigene14840 | ERBB-3 binding protein 1 | EBP1 | AT3G51800 | 8.16 |
| Unigene24435 | Cyclin-dependent kinase B1-1 | CDKB1;1 | AT3G54180 | 12.78 |
| Unigene26214 | Cyclin-dependent kinase B2-2 | CDKB2;2 | AT1G20930 | 8.59 |
| Unigene22462 | Cyclin-dependent kinase C-1 | CDKC;1 | AT5G10270 | 0.30 |
| CL6440.Contig2 | Cyclin-dependent kinase D1;3 | CDKD1;3 | AT1G18040 | 0.13 |
| CL6440.Contig1 | Cyclin-dependent kinase D1;3 | CDKD1;3 | AT1G18040 | 0.11 |
| Unigene23012 | Cyclin H;1 | CYCH;1 | AT5G27620 | 0.07 |
| CL5166.Contig1 | CDK-Subunit 2 | CKS2 | AT2G27970 | 17.22 |
| Unigene25653 | MEI2-like protein 5 | ML5 | AT1G29400 | 4.65 |
| CL6726.Contig5 | Mitotic checkpoint protein BUB3.1 | BUB3.1 | AT3G19590 | 1.69 |
| CL6524.Contig2 | Essential meiotic endonuclease 1B | EME1B | AT2G22140 | 3.60 |
| Unigene26576 | Kinetochore protein SPC24 homolog | SPC24 | AT3G08880 | 7.18 |
| CL1680.Contig2 | Targeting protein for Xklp2 | TPX2 | AT5G15510 | 8.63 |
| Unigene859 | Serine/threonine-protein phosphatase 2A activator | PTPA | AT4G08960 | 4.79 |
| Unigene25162 | Puromycin-sensitive aminopeptidase | MPA1 | AT1G63770 | 0.30 |
| Signalling (16) | | | | |
| Phosphatase and kinase (8) | | | | |
| CL7418.Contig1 | AGC kinase family protein |  | AT4G14350 | 5.25 |
| CL6518.Contig1 | Serine/Threonine Kinase 1 | ASK1 | AT1G10940 | 2.75 |
| CL4952.Contig1 | RNA-binding family protein | RBP | AT5G66010 | 0.23 |
| CL5565.Contig2 | RNA-binding family protein | RBP | AT5G66010 | 0.44 |
| CL2871.Contig3 | ATB' ALPHA | PP2A B subunit | AT5G03470 | 5.35 |
| CL2480.Contig2 | Histidine acid phosphatase |  | AT1G09870 | 0.09 |
| Unigene19516 | Phosphoinositide phosphatase SAC7 | SAC7 | AT3G51460 | 0.54 |
| CL5855.Contig2 | SCP1-like small phosphatase 5 | SSP5 | AT5G11860 | 4.45 |
| Others (8) | | | | |
| CL121.Contig5 | Calmodulin-binding protein |  | AT5G57580 | 8.49 |
| Unigene5788 | Calmodulin-binding protein |  | AT5G39380 | 0.06 |
| Unigene20683 | Annexin 5 | ANN5 | AT1G68090 | 0.31 |
| Unigene24971 | Short root in salt medium 1 | RSA1 | AT2G03150 | 0.28 |
| Unigene23416 | RNA Ligase | ATRNL | AT1G07910 | 5.55 |
| CL1391.Contig1 | SPFH/Band 7/PHB domain containing membrane-associated protein family |  | AT2G03510 | 1.89 |
| Unigene10399 | WUS-interacting protein 1 | WSIP1 | AT1G15750 | 0.12 |
| CL7398.Contig2 | Guanylate-binding protein |  | AT5G46070 | 0.42 |
| Transport (31) | | | | |
| Protein and lipid transport (17) | | | | |
| CL1780.Contig1 | Importin Alpha isoform 1 | IMPA-1 | AT3G06720 | 4.45 |
| Unigene19881 | Signal peptidase | SPC25 | AT2G39960 | 3.87 |
| CL2564.Contig3 | Rab GDP-dissociation inhibitor | GDI | AT5G09550 | 4.49 |
| CL3506.Contig1 | ESCRT-related protein CHMP1B | CHMP1B | AT1G73030 | 3.40 |
| CL1024.Contig1 | Vacuolar protein sorting-associated protein 32-2 | SNF7.1 | AT4G29160 | 11.12 |
| CL7220.Contig4 | Signal recognition particle subunit SRP72 | SRP72 | AT1G67680 | 2.63 |
| Unigene18217 | MPPBETA |  | AT3G02090 | 2.05 |
| CL187.Contig6 | Vacuolar import/degradation | Vid27 | AT3G19240 | 13.03 |
| CL4409.Contig7 | Clathrin, heavy chain | CHC1 | AT3G11130 | 2.37 |
| CL4493.Contig1 | Clathrin adaptor complexes medium subunit family protein |  | AT5G05010 | 2.73 |
| CL5151.Contig1 | ARF-GAP Domain 8 | AGD8 | AT4G17890 | 2.03 |
| CL1107.Contig8 | Coatomer subunit alpha-1 |  | AT1G62020 | 2.85 |
| Unigene8913 | Syntaxin of plants 124 | SYP124 | AT1G61290 | 0.18 |
| Unigene242 | Pleckstrin homology domain protein |  | AT4G11790 | 2.42 |
| Unigene25449 | ABC2 HOMOLOG 16 | ABCA12 | AT5G61700 | 0.41 |
| Unigene24124 | ATP-BINDING CASSETTE F4 | ABCF4 | AT3G54540 | 8.95 |
| CL4205.Contig1 | Calcium-dependent lipid-binding family protein | NTMC2T6.2 | AT3G14590 | 3.94 |
| Ion transport (14) | | | | |
| CL4010.Contig2 | ATP synthase subunit gamma | ATP3 | AT2G33040 | 0.42 |
| CL4159.Contig6 | ATP synthase beta-subunit |  | AT5G08680 | 0.50 |
| Unigene21389 | ATP synthase subunit O | ATP5 | AT5G13450 | 0.34 |
| CL2766.Contig1 | ATP synthase subunit A | VHA-A | AT1G78900 | 0.51 |
| Unigene19960 | Voltage dependent anion channel 2 | VDAC2 | AT5G67500 | 2.88 |
| Unigene29332 | Outer envelope pore protein 16-2 | OEP16-2 | AT4G16160 | 0.47 |
| Unigene16622 | Voltage Dependent Anion Channel 1 | VDAC1 | AT3G01280 | 3.77 |
| Unigene247 | Aquaporin Interactor | AQI | AT4G38220 | 0.25 |
| Unigene5525 | Leucine Zipper-Ef-hand-containing transmembrane protein 2 | LETM2 | AT1G65540 | 0.33 |
| Unigene23858 | SLAH3 |  | AT5G23680 | 9.82 |
| CL5522.Contig1 | ER-type Ca2+-ATPase 1 | ECA1 | AT1G07810 | 0.39 |
| CL2562.Contig2 | SKU5 similar 13 | Sks13 | AT3G13400 | 0.05 |
| CL2562.Contig1 | SKU5 similar 13 | Sks13 | AT3G13400 | 0.12 |
| CL6439.Contig1 | SKU5 similar 14 | Sks14 | AT1G55560 | 0.09 |
| Stress response (79) | | | | |
| Chaperones and foldases (40) | | | | |
| CL2052.Contig5 | Heat shock protein 17.4 | HSP17.4 | AT3G46230 | 5.26 |
| CL4604.Contig2 | 17.6 kDa class II heat shock protein | HSP17.6II | AT5G12020 | 7.45 |
| CL1808.Contig4 | Chaperonin CPN60 | HSP60 | AT3G23990 | 0.19 |
| CL1473.Contig1 | Chaperonin-60alpha | CPN60A | AT2G28000 | 0.17 |
| Unigene17995 | TCP-1/cpn60 chaperonin family protein |  | AT3G03960 | 2.56 |
| Unigene602 | TCP-1/cpn60 chaperonin family protein |  | AT3G02530 | 2.96 |
| CL1635.Contig5 | heat shock protein 70-1 | Hsc70-1 | AT4G24280 | 0.29 |
| CL573.Contig4 | Heat shock protein 70-1 | HSP70-1 | AT5G02500 | 6.03 |
| CL839.Contig3 | Heat shock protein 70-5 | HSC70-5 | AT5G09590 | 0.25 |
| CL717.Contig2 | Heat shock protein 70 | HSC70-2 | AT5G09590 | 0.35 |
| Unigene25906 | Heat Shock Protein 70 | HSP70 | AT3G12580 | 6.92 |
| CL976.Contig2 | Luminal-binding protein 1 | BIP1 | AT5G28540 | 0.22 |
| CL976.Contig4 | Luminal-binding protein 1 | BIP1 | AT5G28540 | 0.29 |
| CL4969.Contig2 | Luminal-binding protein 2 | BIP2 | AT5G42020 | 0.20 |
| CL516.Contig3 | Heat shock protein 91 | HSP91 | AT1G79930 | 0.31 |
| Unigene25305 | Heat shock protein 101 | HSP101 | AT1G74310 | 3.17 |
| Unigene26731 | HSP20-like chaperones | HSP20-like | AT1G54050 | 2.33 |
| CL2341.Contig2 | Carboxylate clamp-tetratricopeptide repeat Proteins | PHOX1 | AT2G25290 | 4.06 |
| CL3199.Contig2 | DNAJ heat shock N-terminal domain-containing protein |  | AT5G18750 | 2.43 |
| Unigene24468 | DNAJ heat shock N-terminal domain-containing protein |  | AT5G53150 | 4.42 |
| CL1654.Contig2 | DNAJ heat shock family protein |  | AT3G08910 | 3.77 |
| Unigene23841 | Chaperone protein dnaJ 6 | ATJ6 | AT5G06910 | 4.38 |
| CL1621.Contig1 | SHEPHERD | SHD | AT4G24190 | 0.38 |
| CL1865.Contig1 | Calnexin 1 | CNX1 | AT5G61790 | 0.14 |
| CL1865.Contig2 | Calnexin 1 | CNX1 | AT5G61790 | 0.27 |
| Unigene22998 | Calnexin 1 | CNX1 | AT5G61790 | 0.13 |
| Unigene22973 | Exostosin domain-containing protein |  | AT3G52220 | 0.37 |
| Unigene24210 | Calreticulin-1 | CRT1a | AT1G56340 | 0.08 |
| Unigene21483 | ERAD-associated E3 ubiquitin-protein ligase HRD1A | HRD1A | AT3G16090 | 0.19 |
| Unigene24163 | ERAD-associated E3 ubiquitin-protein ligase component HRD3A | HRD3A | AT1G18260 | 0.14 |
| Unigene24326 | OS9 | OS9 | AT5G35080 | 0.16 |
| Unigene25210 | Protein disulfide isomerase 6 | PDI6 | AT1G77510 | 0.21 |
| Unigene19345 | Protein disulfide isomerase 10 | PDI10 | AT1G04980 | 0.10 |
| Unigene20582 | PDI-like 1-1 | PDIL1-1 | AT1G21750 | 0.19 |
| Unigene17067 | PDI-like 5-2 | PDIL5-2 | AT1G35620 | 0.21 |
| Unigene9028 | PDIL protein | PDIL 2-1 | AT2G47470 | 0.06 |
| CL1148.Contig2 | FKBP-like peptidyl-prolyl cis-trans isomerase |  | AT1G26550 | 6.28 |
| Unigene25575 | Cyclophilin-like peptidyl-prolyl cis-trans isomerase |  | AT4G32420 | 0.28 |
| Unigene12844 | Peptidyl-prolyl cis-trans isomerase | FKBP15-2 | AT5G48580 | 0.18 |
| Unigene26107 | FK506-binding protein | FKBP15-1 | AT3G25220 | 0.14 |
| Stress response (39) | | | | |
| CL2176.Contig1 | AtRZ-1a | ATRZ-1A | AT3G26420 | 0.31 |
| Unigene6334 | AtRZ-1b | ATRZ-1B | AT1G60650 | 0.17 |
| CL404.Contig1 | AtRZ-1b | ATRZ-1B | AT1G60650 | 0.45 |
| Unigene248 | E3 ubiquitin-protein ligase HOS1 | HOS1 | AT2G39810 | 0.42 |
| Unigene19139 | Cold Shock Domain Protein 3 | CSP3 | AT2G17870 | 4.70 |
| Unigene18891 | Cold shock domain protein 1 | CSDP1 | AT4G36020 | 4.49 |
| CL5306.Contig1 | Cold shock protein 2 | CSP2 | AT4G38680 | 9.08 |
| CL4471.Contig1 | Bifunctional enolase | ENO2 | AT2G36530 | 0.36 |
| Unigene20189 | Heat-stress-associated 32 | HSA32 | AT4G21320 | 4.74 |
| Unigene16547 | Heat intolerant 4 | HIT4 | AT5G10010 | 0.36 |
| CL2531.Contig2 | Responsive to dehydration 21B | RD21B | AT5G43060 | 0.11 |
| CL2531.Contig3 | Responsive to dehydration 21b | RD21B | AT5G43060 | 0.15 |
| Unigene25816 | Stomatal density and distribution 1 | SDD1 | AT1G04110 | 0.16 |
| CL5056.Contig2 | Glutathione s-transferase theta 1 | GSTT1 | AT5G41210 | 2.58 |
| Unigene21103 | Peroxiredoxin-2F | PRXIIF | AT3G06050 | 0.17 |
| CL5903.Contig1 | Peroxiredoxin-II-E |  | AT3G52960 | 0.21 |
| Unigene18320 | Peroxidase 25 | PER25 | AT3G50990 | 0.08 |
| Unigene17566 | 1-Cys peroxiredoxin PER1 | PER1 | AT1G48130 | 7.74 |
| Unigene25549 | Sodium- and lithium-tolerant 1 | SLT1 | AT2G37570 | 0.29 |
| Unigene16352 | Ricin B-like lectin EULS3 | EULS3 | AT2G39050 | 7.73 |
| Unigene26225 | Binding to tomv RNA 1s | BTR1 | AT5G04430 | 2.68 |
| Unigene25711 | Glucosidase 2 subunit beta | PSL4 | AT5G56360 | 0.11 |
| Unigene23746 | TUDOR-SN protein 1 | Tudor1 | AT5G07350 | 2.25 |
| Unigene8459 | Tetraspanin8 | TET8 | AT2G23810 | 0.08 |
| CL679.Contig1 | Late embryogenesis abundant protein | LEA | AT4G36600 | 0.04 |
| CL6827.Contig1 | Late embryogenesis abundant protein | LEA | AT4G13230 | 0.12 |
| Unigene18340 | Late embryogenesis abundant protein | LEA | AT4G13230 | 0.05 |
| CL1922.Contig2 | Late embryogenesis abundant protein | LEA | AT3G02480 | 0.19 |
| Unigene26278 | Late embryogenesis abundant protein | LEA | AT3G02480 | 0.14 |
| Unigene27587 | Late embryogenesis abundant protein | LEA | AT5G66780 | 0.10 |
| Unigene23960 | Late embryogenesis abundant protein | LEA | AT1G72100 | 0.31 |
| CL1922.Contig1 | Late embryogenesis abundant protein | LEA | AT5G38760 | 0.21 |
| Unigene230 | Late embryogenesis abundant protein | UNE15 | AT4G13560 | 0.13 |
| Unigene21627 | Late embryogenesis abundant protein | UNE15 | AT4G13560 | 0.15 |
| Unigene24483 | Development and Cell Death domain protein | DCD | AT5G61910 | 0.37 |
| Unigene24481 | Development and Cell Death domain protein | DCD | AT5G61910 | 0.41 |
| Unigene26212 | Development and Cell Death domain protein | DCD | AT2G32910 | 0.15 |
| Unigene24567 | Development and Cell Death domain protein | DCD | AT2G32910 | 0.28 |
| Unigene24482 | Development and Cell Death domain protein | DCD | AT2G32910 | 0.33 |
|  | **Ubiquitin-related** **proteolysis (36)** | | | |
| Unigene15732 | E3 ubiquitin-protein ligase HOS1 | HOS1 | AT2G39810 | 3.05 |
| Unigene23270 | E3 ubiquitin-protein ligase RING1-like |  | AT3G19950 | 7.91 |
| Unigene24076 | E3 SUMO-protein ligase MMS21 | MMS21 | AT3G15150 | 2.86 |
| CL690.Contig1 | Probable E3 ubiquitin-protein ligase | ARI8 | AT1G65430 | 11.59 |
| CL1941.Contig1 | SKP1-like 3 | SK3 | AT2G25700 | 18.52 |
| CL1620.Contig1 | SKP1-like 12 | SK12 | AT4G34470 | 7.22 |
| CL3643.Contig3 | Cullin 1 | CUL1 | AT4G02570 | 6.55 |
| CL60.Contig1 | Cullin4 | CUL4 | AT5G46210 | 0.24 |
| Unigene22360 | DNA damage-binding protein 1a | DDB1A | AT4G05420 | 0.31 |
| CL6638.Contig1 | Cullin-Associated And Neddylation Dissociated 1 | CAND1 | AT2G02560 | 0.23 |
| CL409.Contig9 | F-box protein |  | AT3G54460 | 3.16 |
| CL2448.Contig1 | COP1 | COP1 | AT2G32950 | 0.28 |
| Unigene18371 | COP9 signalosome complex subunit 8 | CSN8 | AT4G14110 | 2.94 |
| Unigene22706 | COP9 signalosome subunit 4 | CSN4 | AT5G42970 | 0.41 |
| Unigene25625 | SPA1-related 2 | SPA2 | AT4G11110 | 0.35 |
| Unigene21656 | Ubiquitin-protein ligase 3 | UPL3 | AT4G38600 | 3.28 |
| Unigene21653 | Ubiquitin-protein ligase 3 | UPL3 | AT4G38600 | 1.87 |
| Unigene21654 | ubiquitin-protein ligase 3 | UPL3 | AT4G38600 | 4.09 |
| Unigene24560 | pleiotropic regulatory locus 1 | PRL1 | AT4G15900 | 0.34 |
| Unigene16511 | Mos4-associated complex 3a | MAC3A | AT1G04510 | 0.30 |
| CL4766.Contig1 | 26S proteasome regulatory subunit 7 | RPT1A | AT1G53750 | 0.19 |
| CL5070.Contig1 | 26S proteasome regulatory subunit 4 | RPN10 | AT4G38630 | 0.24 |
| Unigene14088 | 26S proteasome regulatory subunit 8 | RPN12A | AT1G64520 | 0.47 |
| Unigene17446 | 26S proteasome regulatory complex |  | AT2G32730 | 0.34 |
| CL1383.Contig1 | 26S proteasome regulatory subunit N1 | RPN1A | AT2G20580 | 0.28 |
| CL5159.Contig1 | 26S proteasome AAA-ATPase subunit | RPT3 | AT5G58290 | 0.44 |
| CL5667.Contig3 | 26S proteasome regulatory subunit 12 | RPN5A | AT5G09900 | 0.26 |
| Unigene18046 | 20S Proteasome Alpha Subunit G1 | PAG1 | AT2G27020 | 0.12 |
| CL3733.Contig2 | 20s proteasome beta subunit G1 | PBG1 | AT1G56450 | 0.36 |
| Unigene14687 | 20S proteasome alpha subunit C1 | PAC1 | AT3G22110 | 0.10 |
| CL6813.Contig2 | 20S proteasome beta subunit | PBF1 | AT3G60820 | 0.06 |
| CL5723.Contig2 | Proteasome Subunit PAB1 | PAB1 | AT1G16470 | 0.11 |
| CL2119.Contig1 | Proteasome alpha subunit F1 | PAF1 | AT5G42790 | 0.24 |
| CL3641.Contig2 | Protease Do-like 7 | DEG7 | AT3G03380 | 0.29 |
| Unigene27034 | Presequence protease 1 | PREP1 | AT3G19170 | 0.18 |
| Unigene22392 | Aspartic proteinase A1 | APA1 | AT1G11910 | 0.13 |
|  | **DNA recombination and repair (19)** | | | |
| Unigene20795 | poly(ADP-ribose) polymerase 1 | PARP1 | AT2G31320 | 0.43 |
| Unigene20693 | DNA double-strand break repair protein |  | AT1G55170 | 0.33 |
| Unigene24320 | Damaged DNA binding 2 | DDB2 | AT5G58760 | 0.43 |
| Unigene16421 | DNA repair protein RAD50 | RAD50 | AT2G31970 | 2.59 |
| Unigene12908 | DNA repair protein RAD50 | RAD50 | AT2G31970 | 2.52 |
| Unigene5825 | RAD50 | RAD50 | AT2G31970 | 2.56 |
| Unigene25505 | RAD4 | RAD4 | AT5G16630 | 2.75 |
| Unigene8753 | DNA repair helicase UVH6 | UVH6 | AT1G03190 | 0.18 |
| Unigene878 | DNA repair endonuclease UVH1 | UVH1 | AT5G41150 | 0.20 |
| Unigene25407 | DNA repair endonuclease UVH1 | UVH1 | AT5G41150 | 0.39 |
| Unigene23448 | Ultraviolet Hypersensitive 3 | UVH3 | AT3G28030 | 0.49 |
| Unigene18137 | Photolyase 1 | PHR1 | AT1G12370 | 0.24 |
| Unigene18136 | Photolyase 1 | PHR1 | AT1G12370 | 0.16 |
| Unigene20835 | 5'-3' exonuclease family protein | FEN1 | AT5G26680 | 3.72 |
| Unigene4 | Formamidopyrimidine-DNA glycosylase | FPG1 | AT1G52500 | 0.23 |
| CL3608.Contig1 | ATP-dependent DNA helicase 2 subunit KU80 | KU80 | AT1G48050 | 0.26 |
| Unigene24121 | Polynucleotide 3'-phosphatase ZDP | ZDP | AT3G14890 | 4.54 |
| Unigene25473 | BRCT domain-containing DNA repair protein |  | AT3G21480 | 5.60 |
| Unigene25474 | BRCT domain-containing DNA repair protein |  | AT3G21480 | 5.11 |
| Metabolism (83) | | | | |
| Glycolytic process and TCA (24) | | | | |
| Unigene25329 | Pyruvate dehydrogenase E1 alpha | PDH-E1a | AT1G01090 | 0.29 |
| Unigene6863 | Pyruvate decarboxylase-2 | PDC2 | AT5G54960 | 0.06 |
| Unigene25949 | Pyruvate dehydrogenase complex E1 Alpha Subunit | E1 ALPHA | AT1G59900 | 0.17 |
| Unigene18093 | Pyruvate dehydrogenase E1 component subunit beta-1 | PDH2 | AT5G50850 | 0.10 |
| Unigene23207 | Cytosolic Enolase | ENO3 | AT2G29560 | 4.45 |
| Unigene24322 | Aldolase superfamily protein | PDE345 | AT2G01140 | 0.04 |
| Unigene24323 | Aldolase superfamily protein | PDE345 | AT2G01140 | 0.15 |
| CL2328.Contig3 | Aldolase superfamily protein | FBA8 | AT3G52930 | 0.27 |
| CL503.Contig5 | Glyceraldehyde-3-phosphate dehydrogenase C-2 | GAPC2 | AT1G13440 | 0.22 |
| Unigene9875 | Glyceraldehyde-3-phosphate dehydrogenase | GAPCP-2 | AT1G16300 | 0.24 |
| Unigene23598 | Pyruvate decarboxylase-2 | PDC2 | AT5G54960 | 0.04 |
| CL3619.Contig1 | Succinate dehydrogenase 1-1 | SDH1-1 | AT5G66760 | 0.31 |
| Unigene26815 | Citrate synthase 4 | ATCS | AT2G44350 | 0.14 |
| Unigene3677 | Citrate synthase 4 | ATCS | AT2G44350 | 0.18 |
| CL1915.Contig1 | ATP citrate lyase family protein | ACL | AT2G20420 | 0.16 |
| Unigene16523 | ATP citrate lyase family protein | ACL | AT2G20420 | 0.42 |
| CL7257.Contig1 | Aconitase 1 | ACO1 | AT4G35830 | 0.13 |
| CL2850.Contig2 | Aconitase 3 | ACO3 | AT2G05710 | 0.16 |
| CL114.Contig2 | Isocitrate dehydrogenase 1 | IDH1 | AT4G35260 | 0.29 |
| CL4784.Contig1 | Isocitrate dehydrogenase V | IDH-V | AT5G03290 | 0.18 |
| CL4329.Contig2 | 2-oxoglutarate dehydrogenase |  | AT5G65750 | 1.61 |
| Unigene18911 | Malate dehydrogenase | MDH | AT3G47520 | 0.19 |
| Unigene21097 | Malate dehydrogenase 1 | MMdh1 | AT1G53240 | 0.05 |
| CL3116.Contig1 | NADP-malic enzyme 3 | NADP-ME3 | AT5G25880 | 0.14 |
| Carbohydrate metabolism (19) | | | | |
| Unigene20588 | Transaldolase 2 | TRA2 | AT5G13420 | 0.12 |
| Unigene15182 | Phosphoglucomutase | PGMP | AT5G51820 | 0.25 |
| CL2217.Contig1 | Phosphoglucomutase 3 | PGM3 | AT1G23190 | 0.27 |
| CL2079.Contig2 | ADPglc-PPase large subunit | APL2 | AT1G27680 | 0.47 |
| Unigene14819 | pectin methylesterase inhibitor |  | AT3G05610 | 0.13 |
| CL1817.Contig1 | Pectate lyase family protein | AT59 | AT1G14420 | 0.14 |
| CL3977.Contig1 | Galactose oxidase |  | AT1G18610 | 0.27 |
| Unigene1326 | beta-galactosidase 15 | BGAL15 | AT1G31740 | 0.15 |
| Unigene23914 | beta-galactosidase 13 | BGAL13 | AT2G16730 | 0.09 |
| Unigene23975 | beta-galactosidase 7 | BGAL7 | AT5G20710 | 0.05 |
| Unigene25833 | beta-fructofuranosidase | BFRUCT1 | AT3G13790 | 0.09 |
| Unigene20671 | beta-fructofuranosidase | BFRUCT4 | AT1G12240 | 0.25 |
| Unigene17382 | Beta-glucosidase |  | AT5G49900 | 0.29 |
| CL2168.Contig1 | Beta-galactosidase 7 |  | AT5G20710 | 0.22 |
| CL6621.Contig1 | Xylose isomerase |  | AT5G57655 | 0.06 |
| Unigene23799 | Glucan endo-1,3-beta-glucosidase 8 |  | AT1G64760 | 0.09 |
| CL3686.Contig1 | fructokinase-5 | Frk | AT4G10260 | 0.21 |
| Unigene17517 | alpha-glucan phosphorylase 1 | PHS1 | AT3G29320 | 0.16 |
| Unigene24304 | Bifunctional dTDP-4-dehydrorhamnose 3,5-epimerase/dTDP-4-dehydrorhamnose reductase | NRS/ER | AT1G63000 | 6.37 |
| Fatty acid metabolism (12) | | | | |
| CL1515.Contig1 | Acyl carrier protein 4 | ACP4 | AT4G25050 | 0.15 |
| Unigene22690 | Acyl-COA oxidase 4 | ACX4 | AT3G51840 | 0.28 |
| Unigene8316 | Acyl-COA Oxidase 1 | ACX1 | AT4G16760 | 0.14 |
| Unigene19589 | 3ketoacyl acyl carrier protein synthase I | KASI | AT5G46290 | 0.27 |
| CL5738.Contig1 | Acetyl Co-Enzyme A carboxylase biotin carboxylase subunit | CAC2 | AT5G35360 | 0.06 |
| CL6392.Contig1 | Peroxisomal fatty acid beta-oxidation multifunctional protein | AIM1 | AT4G29010 | 0.23 |
| Unigene24572 | FAD/NAD(P)-binding oxidoreductase |  | AT5G20080 | 0.11 |
| Unigene16245 | Saccharopine dehydrogenase | SDH | AT5G39410 | 0.33 |
| Unigene22598 | 3-ketoacyl-CoA thiolase 2 | KAT2 | AT2G33150 | 0.17 |
| CL5141.Contig1 | Enoyl-[acyl-carrier-protein] reductase [NADH] | MOD1 | AT2G05990 | 0.05 |
| Unigene25374 | Multifunctional Protein 2 | MFP2 | AT3G06860 | 0.39 |
| CL587.Contig1 | Glycerol kinase | GLI1 | AT1G80460 | 0.42 |
| Amino-acid metabolism (15) | | | | |
| CL1595.Contig1 | Aldehyde dehydrogenase 5f1 | ALDH5F1 | AT1G79440 | 0.24 |
| CL7094.Contig2 | Aldehyde dehydrogenase 2B4 | ALDH2B4 | AT3G48000 | 0.14 |
| CL843.Contig1 | Cobalamin independent methionine Synthase | ATMS1 | AT5G17920 | 0.33 |
| Unigene19518 | Aspartate aminotransferase 3 | ASP3 | AT5G11520 | 0.29 |
| Unigene19014 | 3-phosphoglycerate dehydrogenase | PGDH | AT1G17745 | 0.45 |
| CL5890.Contig2 | Leucyl aminopeptidase 1 | LAP1 | AT2G24200 | 0.10 |
| Unigene24045 | Serine hydroxymethyltransferase 4 | SHM4 | AT4G13930 | 0.42 |
| Unigene23309 | Ketol-acid reductoisomerase |  | AT3G58610 | 0.24 |
| Unigene26238 | Cyclase family protein |  | AT4G35220 | 0.08 |
| CL3925.Contig1 | Phosphoserine aminotransferase | PSAT | AT4G35630 | 0.34 |
| CL7166.Contig1 | Mitochondrial processing peptidase | MPPalpha | AT3G16480 | 2.20 |
| Unigene22007 | prolyl 4-hydroxylase 7 | P4H7 | AT3G28480 | 0.44 |
| Unigene19920 | Serine-type endopeptidase |  | AT5G19580 | 0.14 |
| Unigene8415 | Dihydrolipoyl dehydrogenase 1 | LPD1 | AT1G48030 | 0.32 |
| CL5152.Contig1 | Aminomethyltransferase | GDCST | AT1G11860 | 0.49 |
| Others (13) | | | | |
| Unigene20968 | Chalcone-flavanone isomerase |  | AT5G66230 | 10.86 |
| CL4365.Contig1 | Gamma carbonic anhydrase 2 | GAMMA CA2 | AT1G47260 | 2.25 |
| CL7134.Contig2 | Adenylosuccinate synthase | ADSS | AT3G57610 | 0.21 |
| Unigene6054 | Orotate phosphoribosyltransferase |  | AT3G54470 | 6.79 |
| CL2018.Contig2 | Thiosulfate/3-mercaptopyruvate sulfurtransferase 1 | STR1 | AT1G79230 | 0.44 |
| Unigene24222 | AAA-type ATPase family protein |  | AT4G02480 | 0.39 |
| Unigene19602 | Suppressor of NPH4 2 | SNP2 | AT3G50660 | 0.28 |
| Unigene23129 | Pyrophosphorylase 6 | PPa6 | AT5G09650 | 0.16 |
| Unigene25255 | EMBRYO DEFECTIVE 1467 | EMB1467 | AT5G37510 | 1.74 |
| Unigene8408 | Carbonic anhydrase | CAH1 | AT3G52720 | 0.04 |
| CL4793.Contig1 | Cupin family protein |  | AT2G28680 | 0.33 |
| Unigene21130 | Alpha/beta-hydrolases superfamily protein |  | AT5G41850 | 9.46 |
| CL7276.Contig1 | NADH dehydrogenase | NDB3 | AT4G21490 | 0.33 |
| Function unknown (52) | | | | |
| CL5846.Contig1 | Cytomatrix protein-like protein |  | AT1G19980 | 7. 79 |
| Unigene22752 | coiled-coil protein |  | AT1G16210 | 5.81 |
| Unigene23615 | pentatricopeptide repeat 6 | PPR6 | AT1G77360 | 0.20 |
| CL3028.Contig5 | WD40 repeat-like superfamily protein |  | AT3G15610 | 5.40 |
| Unigene23725 | YTH domain-containing protein | ECT7 | AT1G48110 | 8.80 |
| Unigene22278 | RHD-LIKE2 | RL2 | AT5G45160 | 0.43 |
| CL1724.Contig2 | Expressed protein |  | AT2G24440 | 33.95 |
| CL3575.Contig4 | Uncharacterized protein |  | AT3G19340 | 0.24 |
| CL2342.Contig3 | Uncharacterized protein |  | AT5G19950 | 6.12 |
| CL2338.Contig3 | Uncharacterized protein |  | AT4G17330 | 5.60 |
| Unigene25289 | Uncharacterized protein |  | AT5G07980 | 4.26 |
| Unigene23654 | Uncharacterized protein |  | AT5G22040 | 0.56 |
| CL2781.Contig2 | Uncharacterized protein |  | AT5G62550 | 26.51 |
| Unigene20501 | Uncharacterized protein |  | AT3G08020 | 3.10 |
| CL3171.Contig6 | Uncharacterized protein |  | AT3G58110 | 2.39 |
| CL4381.Contig4 | Uncharacterized protein |  | AT5G53440 | 9.61 |
| Unigene22350 | Uncharacterized protein |  | AT4G18150 | 3.70 |
| Unigene11652 | Uncharacterized protein |  | AT1G16850 | 0.20 |
| Unigene17605 | Uncharacterized protein |  | AT5G11600 | 0.40 |
| Unigene21957 | Uncharacterized protein |  | AT5G62550 | 29.80 |
| Unigene459 | Uncharacterized protein |  | AT3G62300 | 18.57 |
| CL1174.Contig1 | Uncharacterized protein |  | AT5G12950 | 0.20 |
| Unigene23732 | Uncharacterized protein |  | AT1G23170 | 4.02 |
| CL285.Contig7 | Uncharacterized protein |  | AT1G76740 | 20.79 |
| CL5277.Contig1 | Uncharacterized protein |  | AT1G42960 | 0.15 |
| CL6919.Contig1 | Anth |  |  | 19.66 |
| Unigene18 | Anth |  |  | 13.21 |
| Unigene12983 | null |  |  | 4.69 |
| Unigene23905 | Null |  |  | 2.33 |
| Unigene23473 | null |  |  | 0.17 |
| Unigene19843 | Null |  |  | 0.45 |
| Unigene24446 | Null |  |  | 5.06 |
| Unigene9061 | Null |  |  | 0.24 |
| Unigene20589 | Null |  |  | 0.17 |
| CL1767.Contig2 | Null |  |  | 0.06 |
| Unigene21812 | Null |  |  | 0.21 |
| CL4994.Contig2 | Null |  |  | 2.27 |
| Unigene23112 | Null |  |  | 11.69 |
| Unigene22924 | Null |  |  | 4.28 |
| CL808.Contig1 | Null |  |  | 0.41 |
| Unigene16300 | Null |  |  | 0.52 |
| Unigene17017 | Null |  |  | 4.21 |
| Unigene442 | Null |  |  | 4.71 |
| Unigene24445 | Null |  |  | 4.45 |
| Unigene20940 | Null |  |  | 0.06 |
| Unigene21275 | Null |  |  | 0.08 |
| Unigene11353 | Null |  |  | 3.08 |
| CL4414.Contig1 | Null |  |  | 0.09 |
| CL806.Contig3 | Null |  |  | 1.96 |
| CL3737.Contig1 | Null |  |  | 0.27 |
| CL2773.Contig2 | Null |  |  | 0.56 |
| CL1867.Contig1 | Null |  |  | 2.15 |

**Table S4 list of the identified transcription factors**

| RNA-seq ID | Description | TF Family | Function | Reference |
| --- | --- | --- | --- | --- |
| Transcription factor highly expressed in VN (12) | | | | |
| Unigene22395 | CDC5 | MYB-related | Regulation of cell cycle, microRNA transcription | Zhang, S., et al.,2013; Lin, Z., et al., 2007 |
| CL5179.Contig2 | HMG box protein with ARID | ARID |  | Hansen, F.T., et al.,2008 |
| Unigene16954 | STK-related TFs | GeBP |  | Zourelidou, M., et al.,2002 |
| CL1029.Contig2 | ATO | C2H2 | Regulators of gametic cell fate | Moll, C., et al., 2008; |
| Unigene16864 | AGL 66 | MADS | Flowering development |  |
| Unigene5688 | AGL104 | MADS | Pollen maturation and tube growth | Adamczyk, B.J and D.E. Fernandez, 2009 |
| Unigene16424 | BZIP61 | bZIP |  | Droge-Laser, W., et al., 2018; Gibalova et al., 2017 |
| Unigene24793 | Transcription factor-like protein |  |  |  |
| CL1732.Contig1 | TFIIE |  |  |  |
| Unigene19852 | TFIIE |  |  |  |
| Unigene300 | TFIIH subunit H1 |  |  |  |
| Unigene25958 | AIPP3 |  |  |  |
| Transcription factor highly expressed in GN (17) | | | | |
| Unigene23227 | NAP57 | NAC | RNA processing | Schorova, S., et al.,2019; |
| Unigene21795 | STK-related TFs | GeBP |  |  |
| Unigene22672 | STK-related TFs | GeBP |  |  |
| Unigene24387 | STK-related TFs | GeBP |  |  |
| Unigene621 | RBP | C2H2 |  |  |
| CL131.Contig1 | MBS1 | C2H2 | ^1^ O_2_ signalling | Shumbe, L., et al., 2017 |
| CL2063.Contig3 | RING/U-box superfamily | C2H2 |  |  |
| Unigene25241 | PTM | PHD | Retrograde signal pathway | Sun, X., et al., 2011 |
| Unigene5682 | NF-YB11 | NF-YB |  | Mu et al., 2013 |
| CL4468.Contig1 | NF-YC10 | CCAAT-HAP5 | Heat stress | Sato, H., et al., 2014 |
| Unigene24902 | RKD3 | RKD | Cell differentiation | Tedeschi, F., et al., 2017 |
| CL7121.Contig1 | AL3 | Alfin-like | Cell differentiation | Liang, X., et al., 2018 |
| Unigene7122 | AL3 | Alfin-like |  | Liang, X., et al., 2018 |
| CL4663.Contig1 | BIM1 | bHLH | Brassinosteroid signaling, male fertility | Chandler, J.W., et al., 2009; Xing, S., et al., 2013 |
| CL900.Contig3 | AIPP1 |  | RNA processing of intronic heterochromatin containing genes | Duan, C.G., et al., 2017 |
| Unigene24317 | EDM2 |  | As AIPP1 | Duan, C.G., et al., 2017 |
| CL240.Contig3 | GTE8 |  |  |  |
